# Supplementary material for: Anle138b binds predominantly to the central cavity in lipidic Aβ₄₀ fibrils and modulates fibril formation
Source: Nat Commun. 2025 Oct 3;16:8850. doi: 10.1038/s41467-025-64443-6 (PMC12494798; doi:10.1038/s41467-025-64443-6)
Supplement: Supplementary file 1 — Supplementary Information [file 41467_2025_64443_MOESM1_ESM.pdf]

## Supplemental Information

# Anle138b binds predominantly to the central cavity in lipidic A$\beta_{40}$ fibrils and modulates fibril formation.

Mookyoung Han[1], Benedikt Frieg[2], Dirk Matthes[3], Andrei Leonov[1,4], Sergey Ryazanov[1,4], Karin Giller[1], Evgeny Nimerovsky[1], Marianna Stampolaki[1], Kai Xue[1], Kerstin Overkamp[1], Christian Dienemann[5], Dietmar Riedel[6], Armin Giese[4], Stefan Becker[1], Bert L. de Groot[3], Gunnar F. Schröder[2,7], Loren B. Andreas[1*] and Christian Griesinger[1,8*]

1. Department of NMR-Based Structural Biology, Max Planck Institute for Multidisciplinary Sciences, Göttingen, Germany.

2. Ernst-Ruska Centre for Microscopy and Spectroscopy with Electrons, ER-C-3 Structural Biology, Forschungszentrum Jülich, Jülich, Germany

3. Department of Theoretical and Computational Biophysics, Max Planck Institute for Multidisciplinary Sciences; Göttingen, Germany.

4. MODAG GmbH, Mikroforum Ring 3, 55234, Wendelsheim, Germany

5. Department of Molecular Biology, Max Planck Institute for Multidisciplinary Sciences, Göttingen, Germany.

6. Laboratory of Electron Microscopy, Max-Planck-Institute for Multidisciplinary Sciences, Göttingen, Germany.

7. Physics Department, Heinrich Heine University Düsseldorf; Düsseldorf, Germany

8. Cluster of Excellence "Multiscale Bioimaging: From Molecular Machines to Networks of Excitable Cells" (MBExC), University of Göttingen; Göttingen, Germany.

* Correspondence and requests for materials should be addressed to Loren B. Andreas (land@mpinat.mpg.de) and Christian Griesinger (cigr@mpinat.mpg.de).

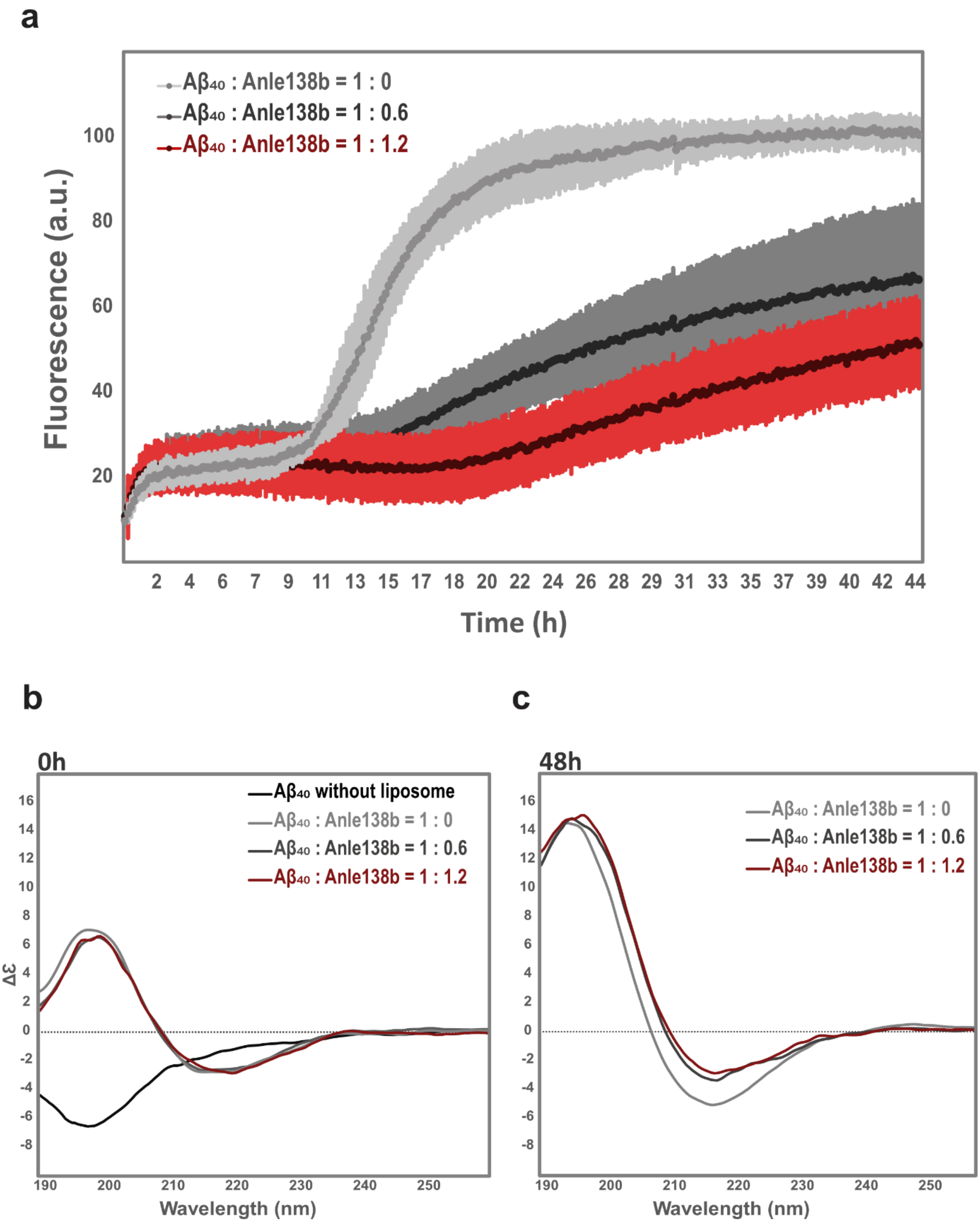
a
Aβ40 : Anle138b = 1 : 0
Aβ40 : Anle138b = 1 : 0.6
Aβ40 : Anle138b = 1 : 1.2
Fluorescence (a.u.)
Time (h)
b
0h
Aβ40 without liposome
Aβ40 : Anle138b = 1 : 0
Aβ40 : Anle138b = 1 : 0.6
Aβ40 : Anle138b = 1 : 1.2
Δε
Wavelength (nm)
c
48h
Aβ40 : Anle138b = 1 : 0
Aβ40 : Anle138b = 1 : 0.6
Aβ40 : Anle138b = 1 : 1.2
Wavelength (nm)

**Supplementary Figure 1 | Anle138b modulates L1 A$\beta_{40}$ fibril formation and secondary structure under the pre-treatment condition.**

**a.** The ThT fluorescence assay shows the effect of increasing concentrations of anle138b on A$\beta_{40}$ fibril formation. The control fibrils (anle138b: A$\beta_{40}$ molar ratio (SMPR) = 0) are shown in light gray, the intermediate concentration (SMPR = 0.6) in dark gray, and the high concentration (SMPR = 1.2) in red. At the highest concentration, anle138b extends the lag phase by approximately 8–10 hours and reduces the overall ThT fluorescence intensity by 40–60%. Fluorescence intensity is shown in arbitrary units (a.u.). Curves show mean fluorescence, with shaded areas indicating mean ± s.d. (n = 6 biological replicates, each from independently prepared A$\beta_{40}$ samples purified from separate expression batches). **b.** Circular dichroism (CD) spectra acquired at 0 hours show β-sheet formation in all conditions (SMPR = 0, 0.6, and 1.2; light gray, dark gray, red). No significant differences were observed between the formation of the β-sheet in the presence or absence of anle138b. **c.** CD spectra after 48 hours of incubation reveal strong β-sheet content in the control fibrils (light gray), while fibrils treated with anle138b (dark gray for SMPR = 0.6, red for SMPR = 1.2) exhibit reduced β-sheet formation. Source data are provided as a Source Data file.

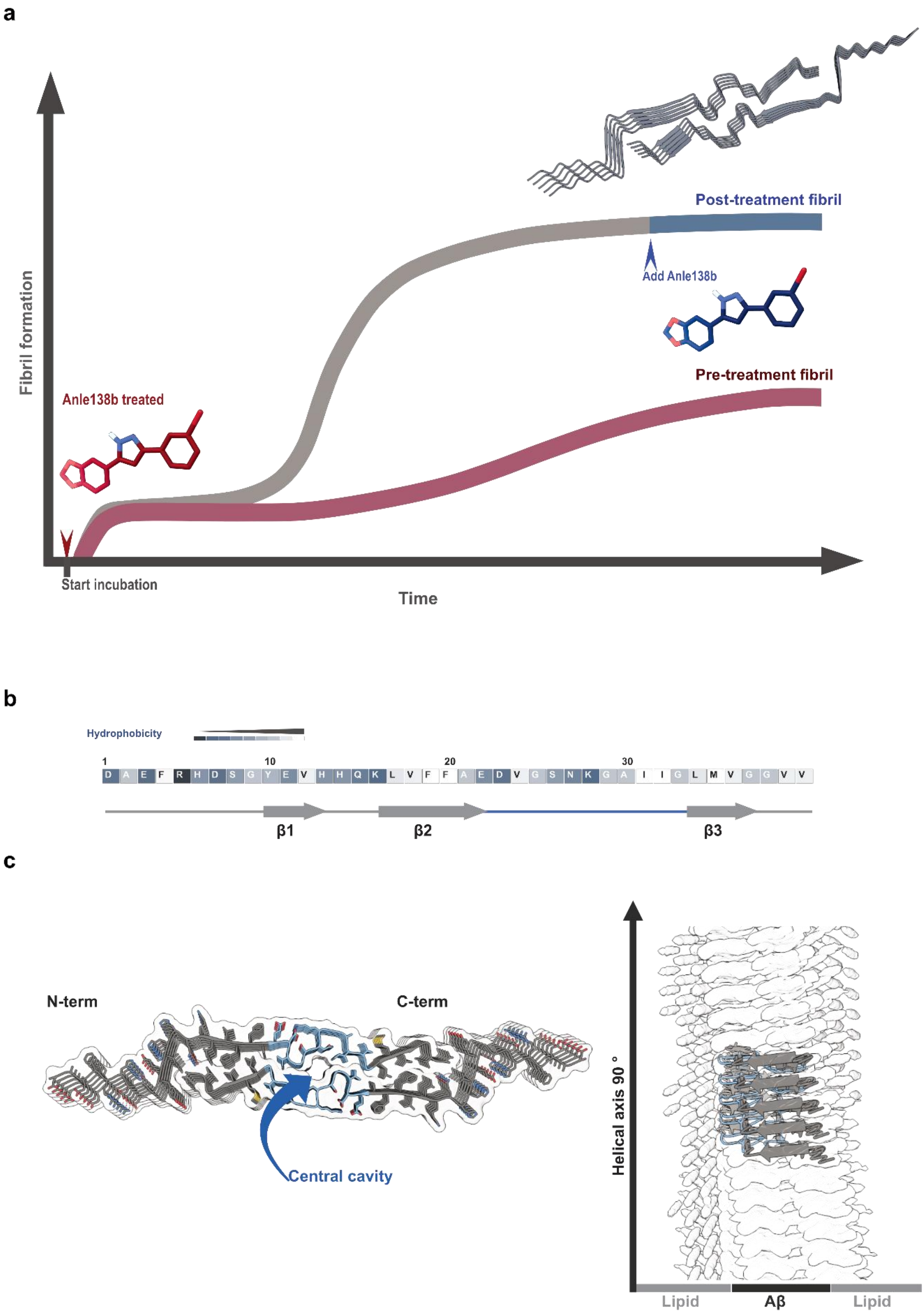
a
Fibril formation
Time
Start incubation
Anle138b treated
Post-treatment fibril
Add Anle138b
Pre-treatment fibril
b
Hydrophobicity
1
10
20
30
D A E F R H D S G Y E V H H Q K L V F F A E D V G S N K G A I I G L M V G G V V
β1
β2
β3
c
N-term
C-term
Central cavity
Helical axis 90 °
Lipid
Aβ
Lipid

**Supplementary Figure 2 | Schematic illustration of experiments investigating the binding sites of anle138b on L1 A$\beta_{40}$ fibrils.**

**a.** Schematic illustration of L1 A$\beta_{40}$ fibril formation under pre-treatment and post-treatment conditions. In the post-treatment condition, anle138b (blue) is added after fibril formation. In the pre-treatment condition, fibrils form in the presence of anle138b (red), which extends the lag phase and reduces the final fibril amount. The two arrows indicate the time points at which anle138b was administered during the fibril formation process. Blue represents the post-treatment condition, and red represents the pre-treatment condition. This schematic provides a conceptual overview of the experimental timeline and administration of anle138b, complementing the corresponding kinetic data shown in Supplementary Fig. 1a and Fig. 1c. **b.** Amino acid sequence and secondary structure of the L1 A$\beta_{40}$ fibril. Hydrophobicity is color-coded using a gradient from dark blue (hydrophilic) to white (hydrophobic), based on the Eisenberg scale[65]. The loop region (Ala21–Gly33), highlighted in blue, contributes to the formation of the central cavity between the two protofilaments. **c.** Structure of the L1 A$\beta_{40}$ fibril based on cryo-EM data. Left: Top view of the fibril showing the loop region (blue); the blue arrow indicates the central cavity. Right: Side view highlighting the periodic alignment of rod-shaped lipid densities along the fibril axis, as visualized in the cryo-EM map.

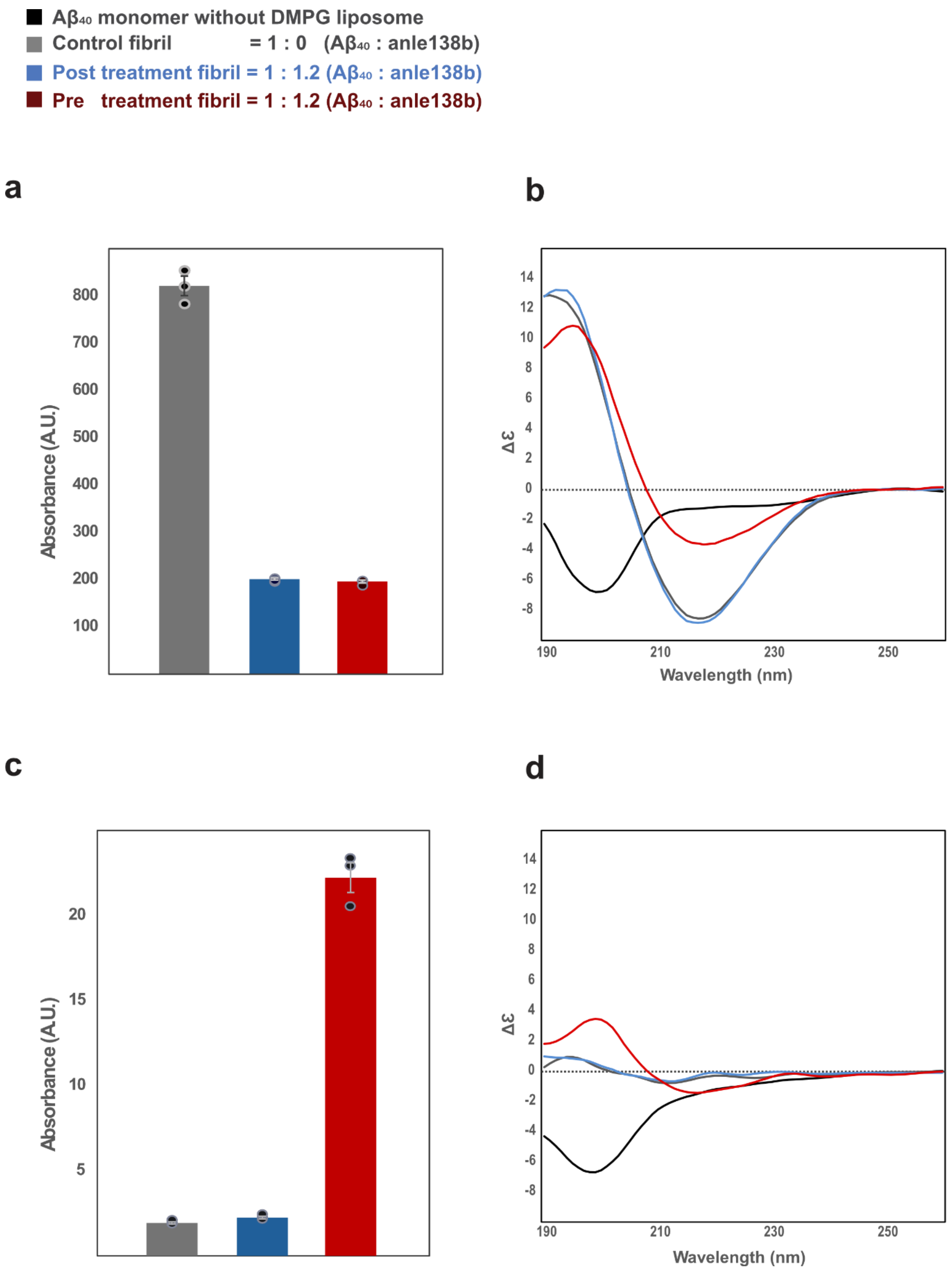
Aβ40 monomer without DMPG liposome
Control fibril = 1 : 0 (Aβ40 : anle138b)
Post treatment fibril = 1 : 1.2 (Aβ40 : anle138b)
Pre treatment fibril = 1 : 1.2 (Aβ40 : anle138b)
a
b
c
d
Absorbance (A.U.)
Absorbance (A.U.)
Δε
Δε
Wavelength (nm)
Wavelength (nm)

**Supplementary Figure 3 | Effects of anle138b on L1 A$\beta_{40}$ fibril formation and structural interaction.**

The results shown here were obtained from the same sample sets used for cryo-EM (Fig. 2) as well as in negative-stain EM, 1D ($^{1}$H) $^{15}$N CP NMR experiments (Fig. 1b, c), 2D $^{13}$C$^{13}$C-DARR spectra (Supplementary Figs. 15, 16), and 2D (H)NCA spectra (Supplementary Fig 5).

**a.** ThT fluorescence assay of L1 A$\beta_{40}$ fibrils prepared under three different conditions. The control sample contains(gray) in the absence of anle138b. In the post-treatment condition (blue; anle138b: A$\beta_{40}$ molar ratio (SMPR) = 1.2) and in the pre-treatment condition (red; SMPR = 1.2). **b.** CD spectra of L1 A$\beta_{40}$ fibrils under the same conditions as in **a**. The control (gray) shows characteristic β-sheet content. The post-treatment condition (blue) exhibits a spectrum similar to the control, indicating that the β-sheet structure is largely preserved. In contrast, the pre-treatment condition (red), where anle138b was present during fibril formation, shows less β-sheet content. A$\beta_{40}$ monomer without DMPG liposome (black) **c.** ThT fluorescence assay of the supernatant obtained after ultracentrifugation of the samples prepared as in panel a. In the pre-treatment condition (red), ThT fluorescence was detected in the supernatant, suggesting the presence of non-fibrillar aggregates remaining in solution. **d.** CD spectra of the same supernatant samples analyzed in panel c. The control (gray) and post-treatment (blue) conditions show flat spectra with no significant secondary structure signals, while the pre-treatment condition (red) displays a β-strand-like spectral pattern, indicating the presence of β-strand structures in the non-fibrillar species within the supernatant. The CD spectrum of A$\beta_{40}$ monomer without DMPG liposome is shown in black. ThT fluorescence is reported in arbitrary units (a.u.); CD spectra are plotted as Δε. Bars in a and c show mean ± s.e.m. with individual data points overlaid (n = 3, technical replicates from a single sample). Source data are provided as a Source Data file.

a

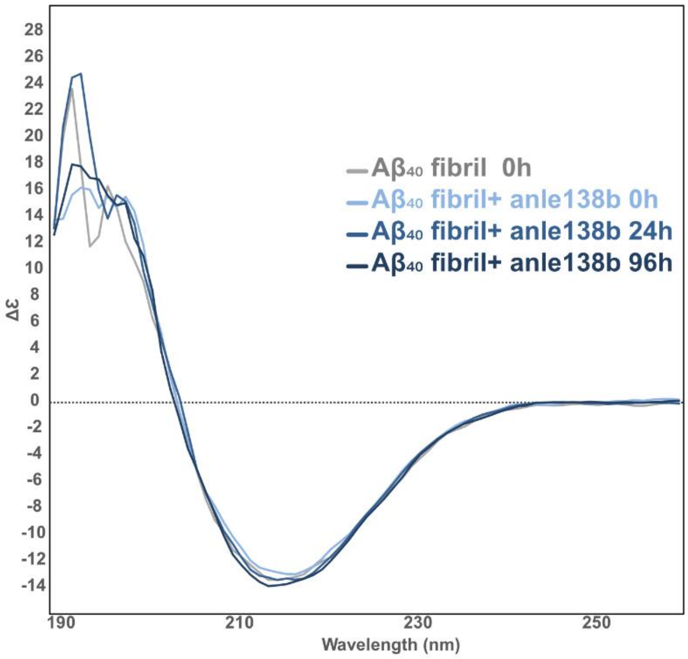

Aβ40 fibril 0h
Aβ40 fibril+ anle138b 0h
Aβ40 fibril+ anle138b 24h
Aβ40 fibril+ anle138b 96h
Δε
28
26
24
22
20
18
16
14
12
10
8
6
4
2
0
-2
-4
-6
-8
-10
-12
-14
190
210
230
250
Wavelength (nm)


b

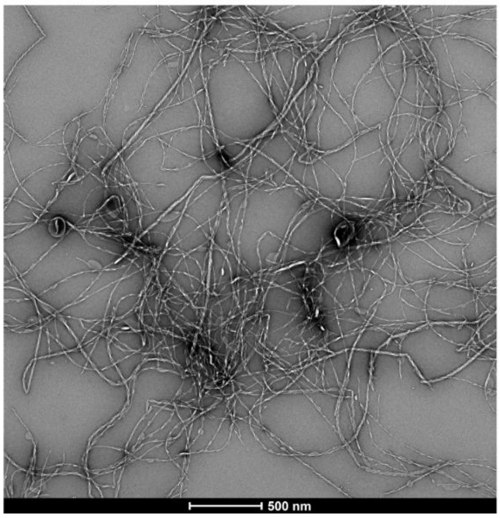

500 nm


c

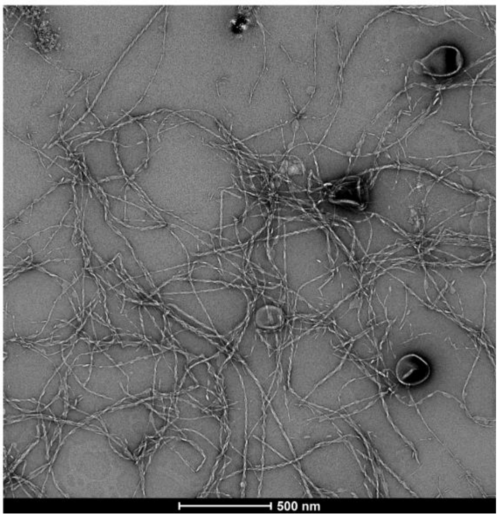

500 nm

**Supplementary Figure 4 | CD spectroscopy and negative-stain EM analysis of L1 A$\beta_{40}$ fibrils under post-treatment condition (anle138b: A$\beta_{40}$ molar ratio (SMPR) = 1.2).**

The secondary structure of L1 A$\beta_{40}$ fibrils treated with anle138b was assessed by CD spectroscopy under post-treatment conditions, following incubation at 37 °C for up to 96 hours. **a.** CD spectra of L1 A$\beta_{40}$ fibrils at various time points after anle138b addition: untreated (gray, 0 h), immediately after treatment (light blue, 0 h), 24 h (blue), and 96 h (dark blue). Source data are provided as a Source Data file. **b, c.** Negative-stain EM images of L1 A$\beta_{40}$ fibrils at 0 h and 96 h after anle138b treatment.

**a**

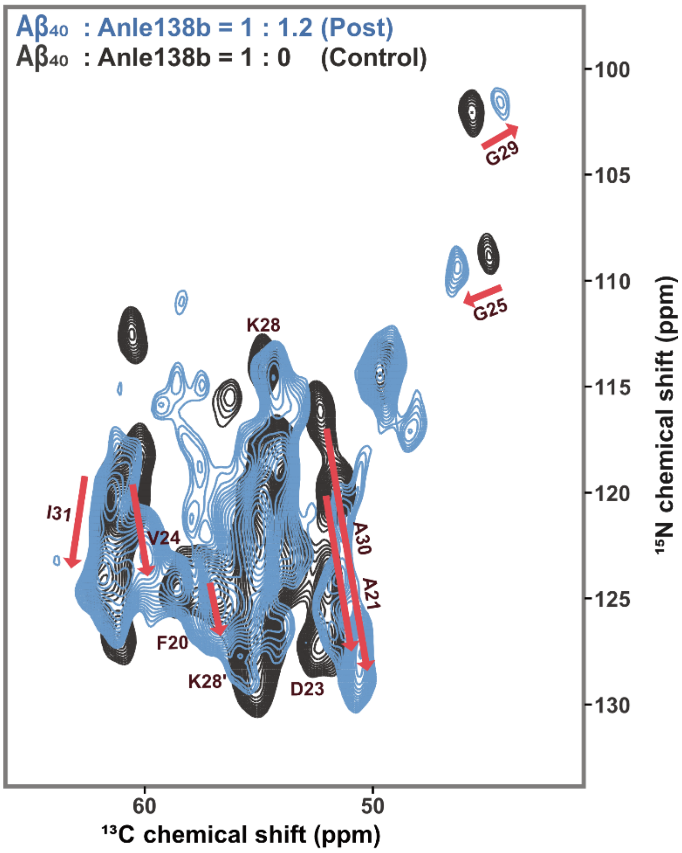
Aβ40 : Anle138b = 1 : 1.2 (Post)
Aβ40 : Anle138b = 1 : 0 (Control)
G29
G25
K28
I31
V24
A30
A21
F20
K28'
D23
100
105
110
115
120
125
130
15N chemical shift (ppm)
60
50
13C chemical shift (ppm)

**b**

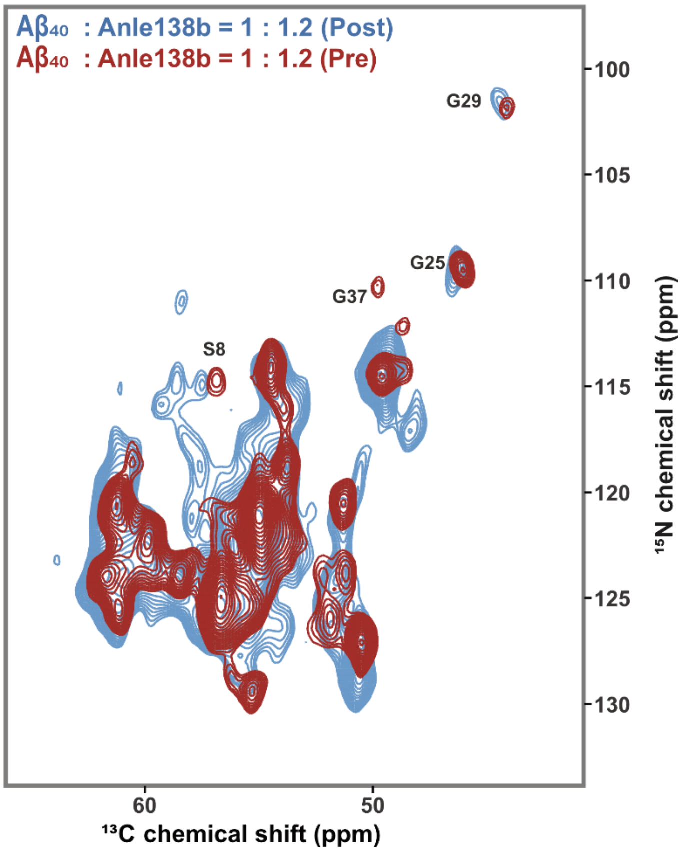
Aβ40 : Anle138b = 1 : 1.2 (Post)
Aβ40 : Anle138b = 1 : 1.2 (Pre)
G29
G25
G37
S8
100
105
110
115
120
125
130
15N chemical shift (ppm)
60
50
13C chemical shift (ppm)

**Supplementary Figure 5 | Superimposed 2D (H)NCA spectra of L1 A$\beta_{40}$ fibrils under control, pre-treatment, and post-treatment conditions.**

The spectra were obtained from the same sample sets utilized in cryo-EM (Fig. 2, Supplementary Figs. 6, 7), negative-stain EM, and solid-state NMR experiments (Fig. 1b, c, Supplementary Figs. 15, 16). **a.** Superimposed 2D (H)NCA spectra of L1 A$\beta_{40}$ fibrils. The blue spectrum represents fibrils treated with anle138b after fibril formation (post-treatment condition; anle138b: A$\beta_{40}$ molar ratio (SMPR) =1.2, ns = 64), while the black spectrum corresponds to fibrils formed in the absence of anle138b (control condition; ns = 64). Chemical shift perturbations induced by anle138b treatment are indicated by pink arrows. **b.** Superimposed 2D (H)NCA spectra comparing the pre- and post-treatment conditions. The red spectrum represents fibrils formed in the presence of anle138b during aggregation (pre-treatment; SMPR 1.2, ns = 192), while the blue spectrum corresponds to the post-treatment condition (SMPR 1.2, ns = 64).

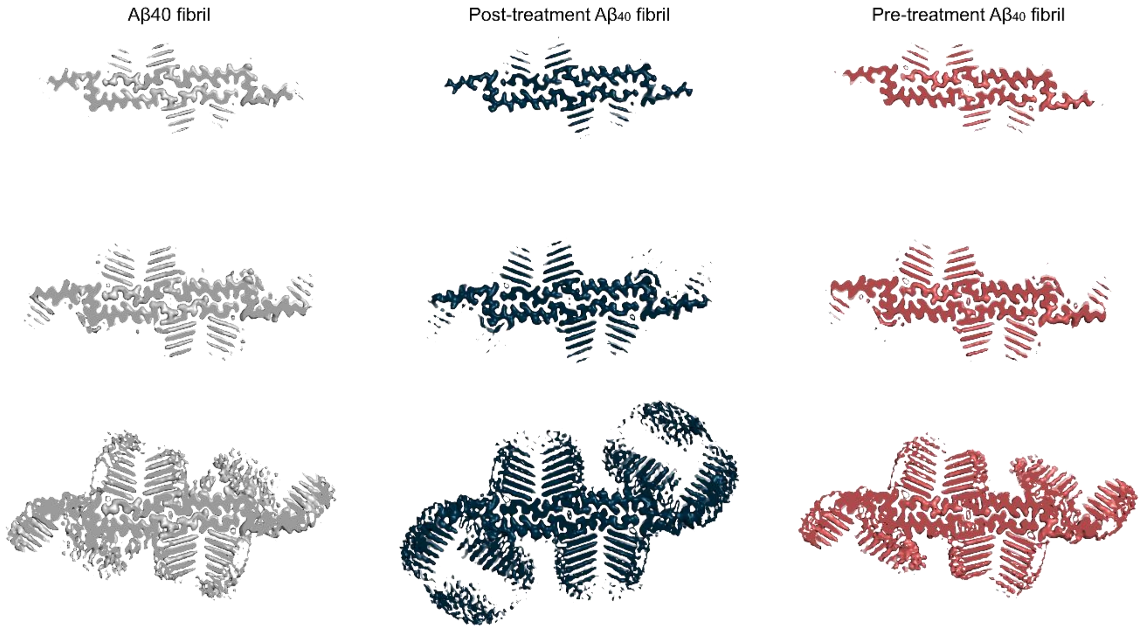


**Supplementary Figure 6 | Sharpened high-resolution maps at different iso-surface levels, L1 Aβ$_{40}$ fibrils.**

From the top to the bottom, the iso-surface level threshold decreases such that additional low-resolution features become visible.

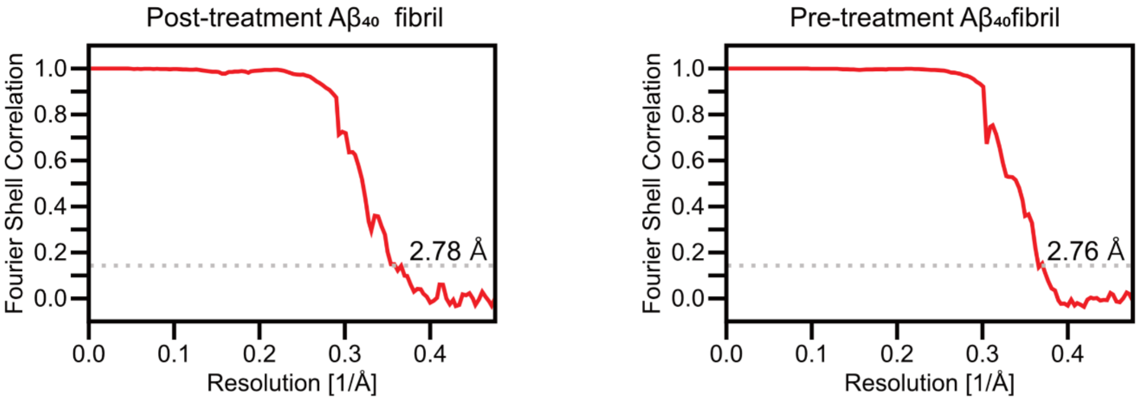


**Supplementary Figure 7 | Fourier shell correlation curves.**

Masked-corrected (z-percentage is 0.1) Fourier shell correlation (FSC) curves. The final resolution is shown in the plot and was estimated from the value of the FSC curve for two separately refined masked half-maps at 0.143 (red line).

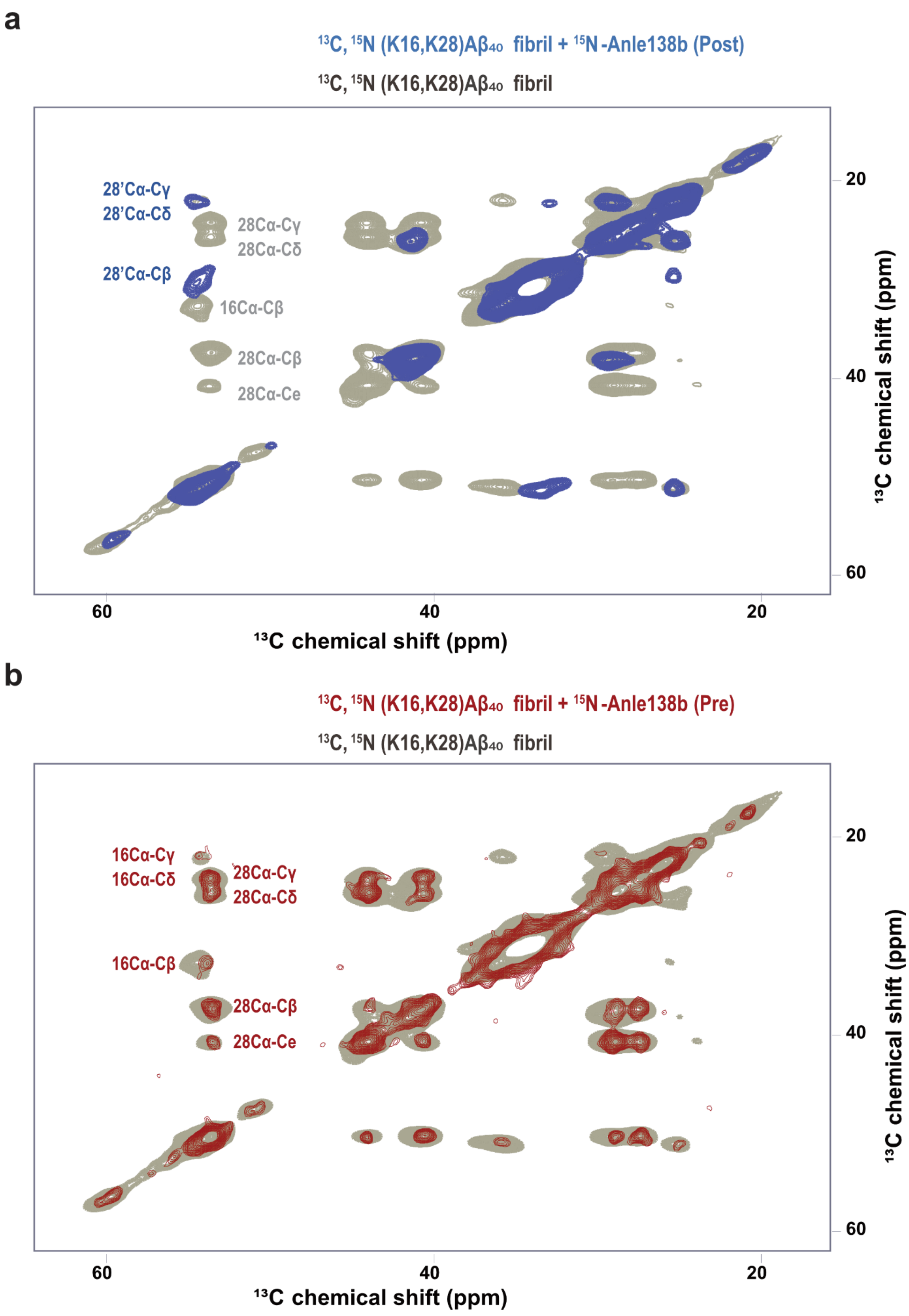

a
13C, 15N (K16,K28)Aβ40 fibril + 15N-Anle138b (Post)
13C, 15N (K16,K28)Aβ40 fibril
28'Cα-Cγ
28'Cα-Cδ
28Cα-Cγ
28Cα-Cδ
28'Cα-Cβ
16Cα-Cβ
28Cα-Cβ
28Cα-Ce
20
40
60
60
40
20
13C chemical shift (ppm)
13C chemical shift (ppm)
b
13C, 15N (K16,K28)Aβ40 fibril + 15N-Anle138b (Pre)
13C, 15N (K16,K28)Aβ40 fibril
16Cα-Cγ
16Cα-Cδ
28Cα-Cγ
28Cα-Cδ
16Cα-Cβ
28Cα-Cβ
28Cα-Ce
20
40
60
60
40
20
13C chemical shift (ppm)
13C chemical shift (ppm)

**Supplementary Figure 8 | 2D $^{13}C^{13}C$-DARR spectra of L1 A$\beta_{40}$ fibrils selectively labeled with $^{13}C$, $^{15}N$ at Lys16 and Lys28.**

**a.** 2D $^{13}C^{13}C$-DARR spectrum acquired at 265 K and 850 MHz with a mixing time of 20 ms. The blue spectrum corresponds to the post-treatment condition. **b.** The red spectrum represents the pre-treatment condition. In both panels, the gray spectrum represents the control condition.

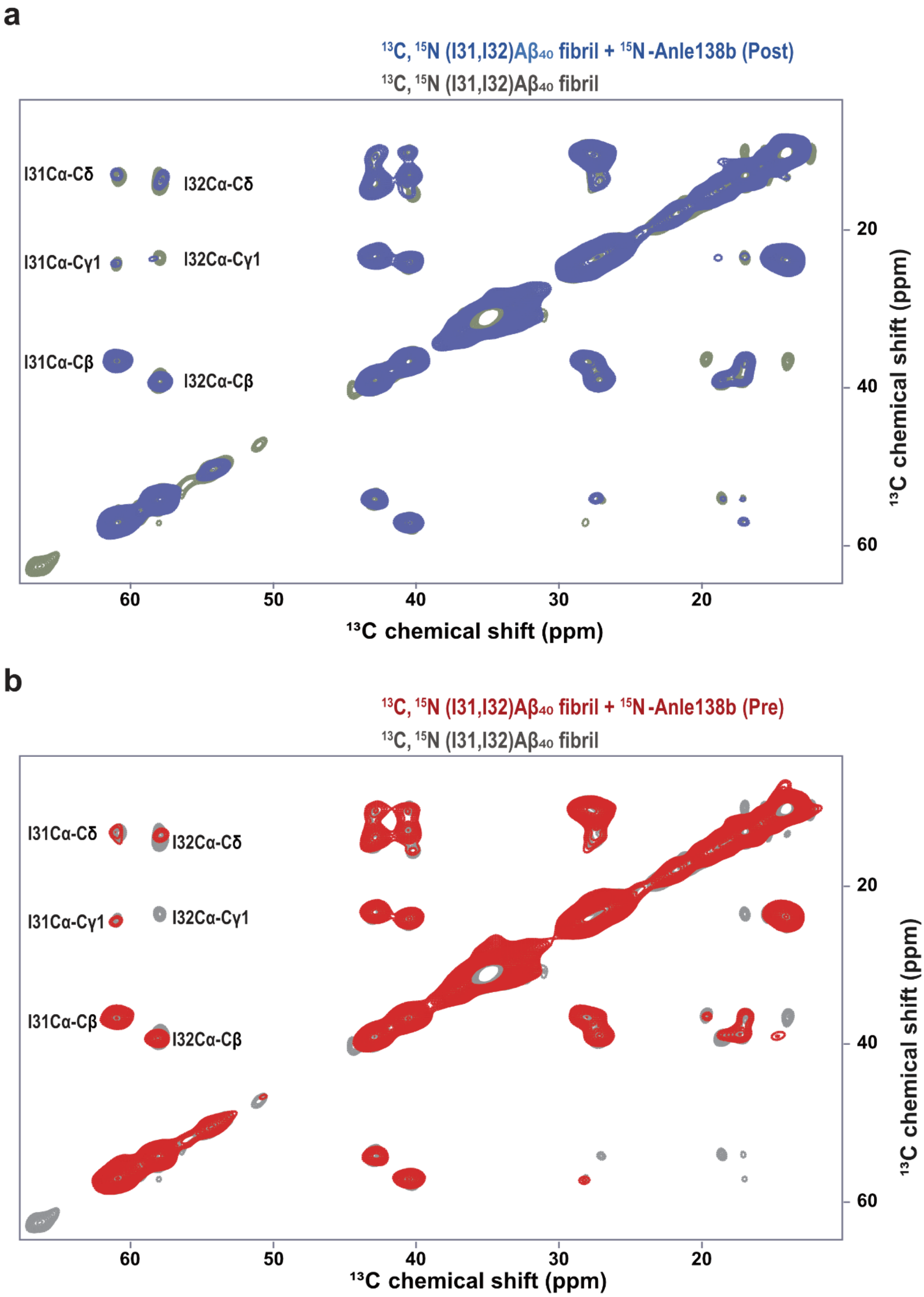
a
13C, 15N (I31,I32)Aβ40 fibril + 15N-Anle138b (Post)
13C, 15N (I31,I32)Aβ40 fibril
I31Cα-Cδ
I32Cα-Cδ
I31Cα-Cγ1
I32Cα-Cγ1
I31Cα-Cβ
I32Cα-Cβ
20
40
60
13C chemical shift (ppm)
60
50
40
30
20
13C chemical shift (ppm)
b
13C, 15N (I31,I32)Aβ40 fibril + 15N-Anle138b (Pre)
13C, 15N (I31,I32)Aβ40 fibril
I31Cα-Cδ
I32Cα-Cδ
I31Cα-Cγ1
I32Cα-Cγ1
I31Cα-Cβ
I32Cα-Cβ
20
40
60
13C chemical shift (ppm)
60
50
40
30
20
13C chemical shift (ppm)

**Supplementary Figure 9 | 2D $^{13}C^{13}C$-DARR spectra of L1 A$\beta_{40}$ fibrils selectively labeled with $^{13}C$, $^{15}N$ at Ile31 and Ile32.**

**a.** 2D $^{13}C^{13}C$-DARR spectrum acquired at 265 K and 850 MHz with a mixing time of 20 ms. The blue spectrum corresponds to the post-treatment condition. **b.** The red spectrum represents the pre-treatment condition. In both panels, the gray spectrum represents the control condition.

**a**

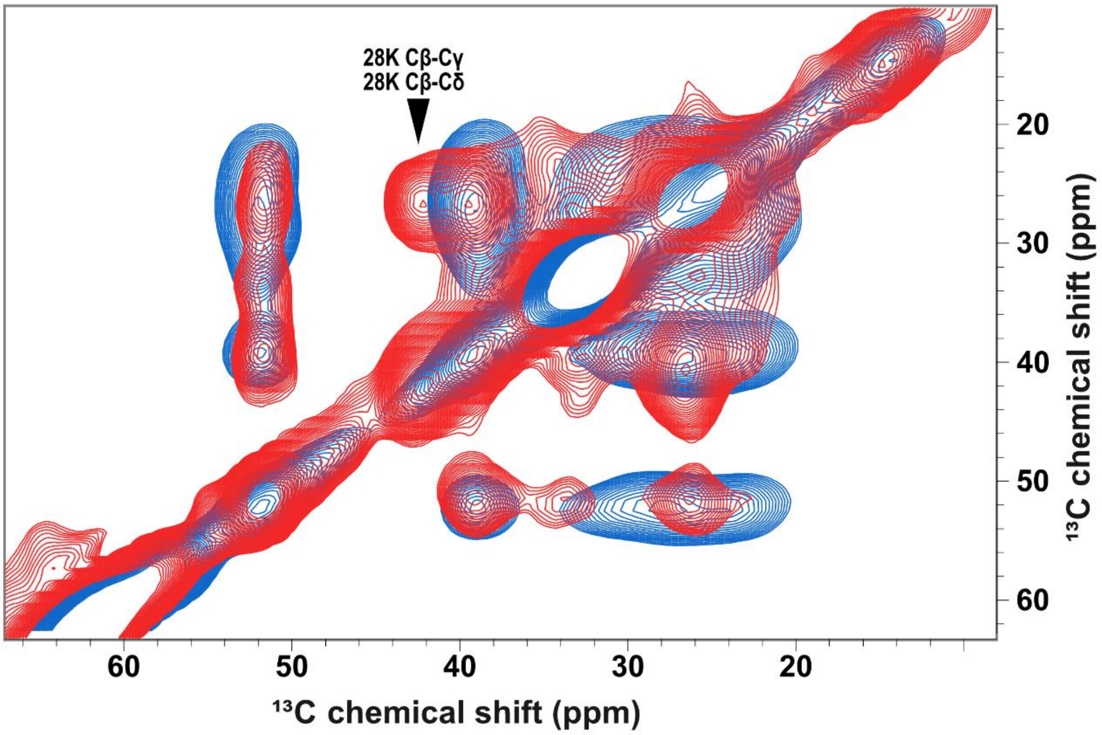
28K Cβ-Cγ
28K Cβ-Cδ
20
30
40
50
60
13C chemical shift (ppm)
60
50
40
30
20
13C chemical shift (ppm)

**b**

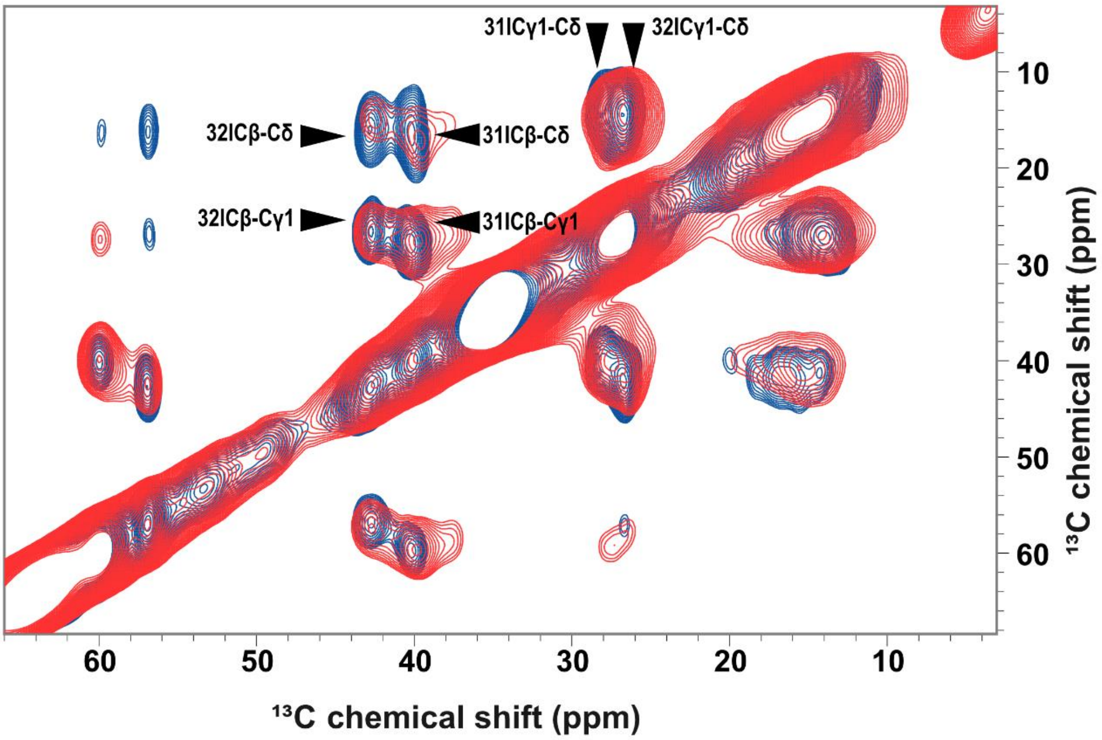
31ICγ1-Cδ
32ICγ1-Cδ
32ICβ-Cδ
31ICβ-Cδ
32ICβ-Cγ1
31ICβ-Cγ1
10
20
30
40
50
60
13C chemical shift (ppm)
60
50
40
30
20
10
13C chemical shift (ppm)

**Supplementary Figure 10 | 2D $^{13}C^{13}C$- DARR and RFDR spectra of selectively labeled L1 A$\beta_{40}$ fibrils used for DNP experiments.**

**a**. 2D $^{13}C^{13}C$–DARR spectra acquired at 100 K and 600 MHz with a mixing time of 50 ms under DNP conditions. Fibrils were selectively labeled with $^{13}C$, $^{15}N$ at Lys16 and Lys28. The blue spectrum represents the post-treatment condition, in which anle138b was added after fibril formation. The red spectrum corresponds to the pre-treatment condition, where anle138b was present during fibril formation. **b.** 2D $^{13}C^{13}C$–RFDR spectra acquired under the same experimental conditions, with a mixing time of 2.6 ms. Fibrils were selectively labeled with $^{13}C$, $^{15}N$ at Ile31 and Ile32. The blue spectrum corresponds to the post-treatment condition, and the red spectrum to the pre-treatment condition. Peak assignments include Cβ–Cγ and Cβ–Cδ correlations of Lys28 (**a**), and Cβ–Cγ1 and Cγ1–Cδ correlations of Ile31 and Ile32 (**b**). Arrows indicate assigned peaks.

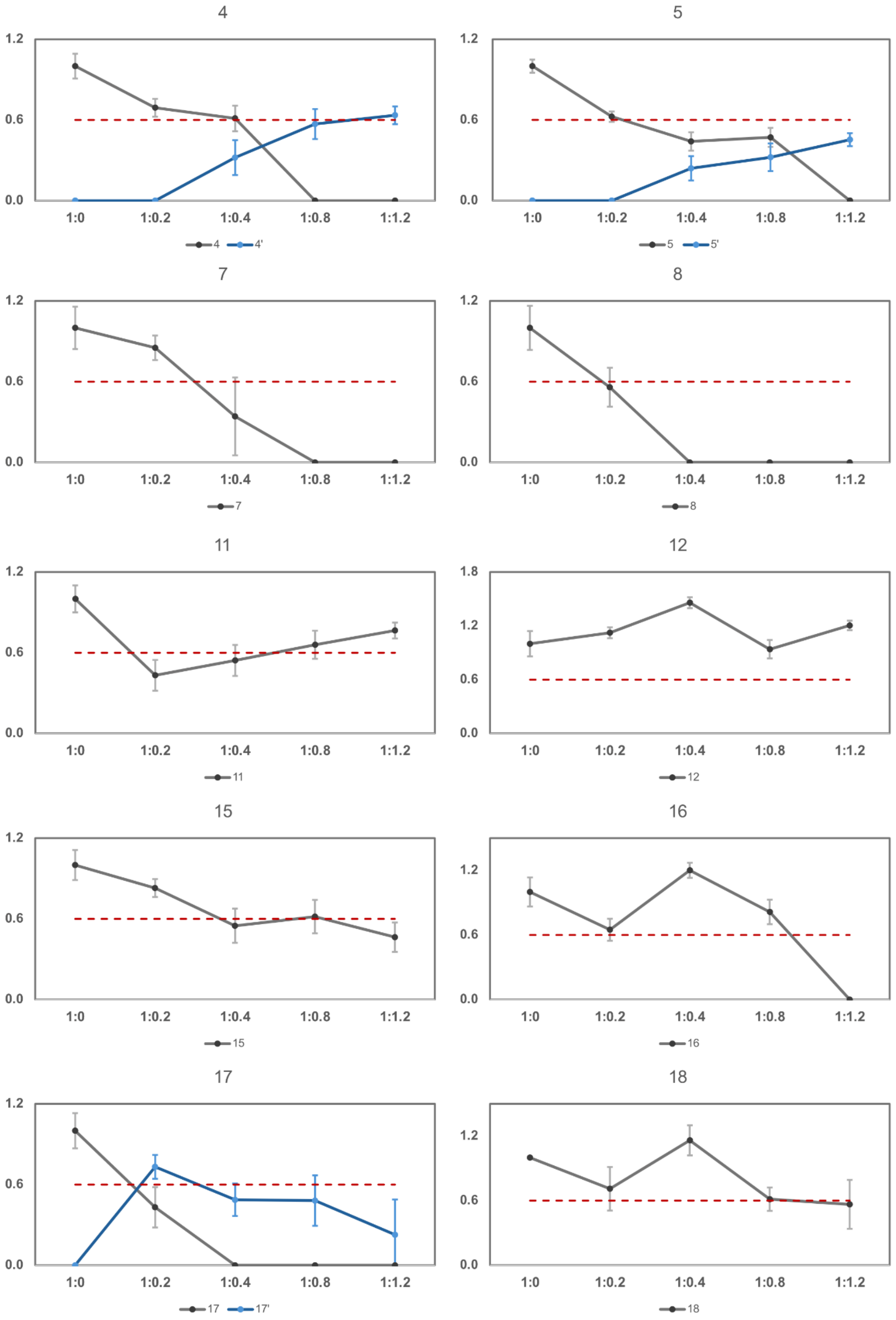
4
1.2
0.6
0.0
1:0
1:0.2
1:0.4
1:0.8
1:1.2
4
4'
5
1.2
0.6
0.0
1:0
1:0.2
1:0.4
1:0.8
1:1.2
5
5'
7
1.2
0.6
0.0
1:0
1:0.2
1:0.4
1:0.8
1:1.2
7
8
1.2
0.6
0.0
1:0
1:0.2
1:0.4
1:0.8
1:1.2
8
11
1.2
0.6
0.0
1:0
1:0.2
1:0.4
1:0.8
1:1.2
11
12
1.8
1.2
0.6
0.0
1:0
1:0.2
1:0.4
1:0.8
1:1.2
12
15
1.2
0.6
0.0
1:0
1:0.2
1:0.4
1:0.8
1:1.2
15
16
1.2
0.6
0.0
1:0
1:0.2
1:0.4
1:0.8
1:1.2
16
17
1.2
0.6
0.0
1:0
1:0.2
1:0.4
1:0.8
1:1.2
17
17'
18
1.2
0.6
0.0
1:0
1:0.2
1:0.4
1:0.8
1:1.2
18

**Supplementary Figure 11 | Titration-dependent intensity changes indicating slow exchange binding of anle138b to residues 4–18 of L1 A$\beta_{40}$ fibrils.**

Signal intensity ratios (I_ratio) were monitored for selected residues of L1 A$\beta_{40}$ as a function of increasing anle138b molar ratios (anle138b: A$\beta_{40}$ molar ratio (SMPR) = 0 to 1.2) under post-treatment conditions. The gray curves represent normalized intensities of original peaks (I_free) observed at SMPR 0, and their changes upon ligand titration (I_bound). The blue curves indicate new peaks indicative of slow exchange. The dashed red line marks the 0.6 intensity threshold used as a reference for qualitative comparison. I_ratio = I_bound / I_free, where I_free refers to the peak intensity in the absence of anle138b, and I_bound refers to the intensity at each titration point. Error bars indicate errors estimated from the signal-to-noise ratio of the NMR spectra. Source data are provided as a Source Data file.

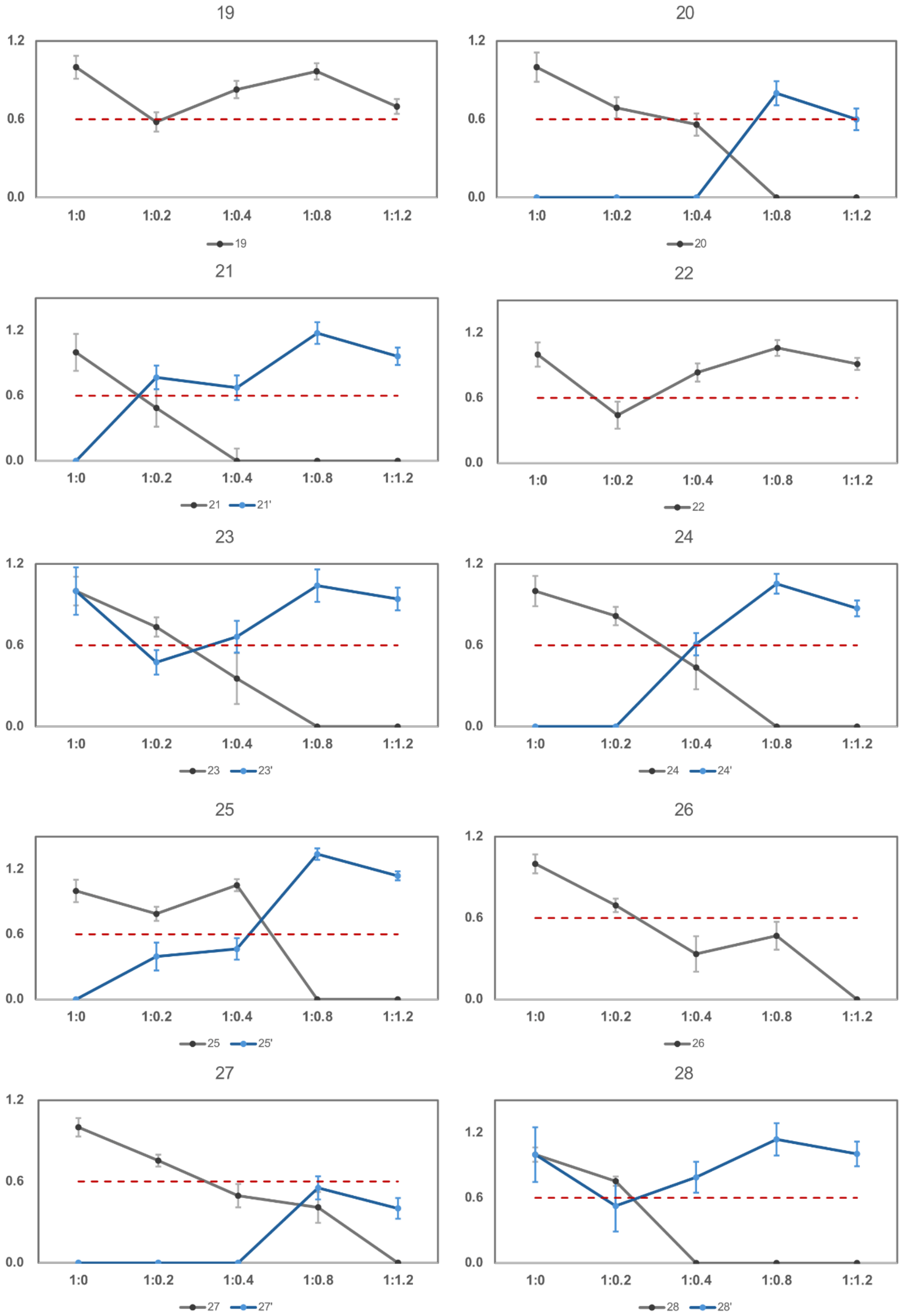
19
1.2
0.6
0.0
1:0
1:0.2
1:0.4
1:0.8
1:1.2
19
20
20
21
21
21'
22
22
23
23
23'
24
24
24'
25
25
25'
26
26
27
27
27'
28
28
28'

**Supplementary Figure 12 | Titration-dependent intensity changes indicating slow exchange binding of anle138b to residues 19–28 of L1 Aβ$_{40}$ fibrils.**

Signal intensity ratios (I_ratio) were monitored for selected residues of L1 Aβ$_{40}$ as a function of increasing anle138b molar ratios (anle138b: Aβ$_{40}$ molar ratio (SMPR) = 0 to 1.2) under post-treatment conditions. The gray curves represent normalized intensities of original peaks (I_free) observed at SMPR 0, and their changes upon ligand titration (I_bound). The blue curves indicate new peaks indicative of slow exchange. The dashed red line marks the 0.6 intensity threshold used as a reference for qualitative comparison. I_ratio = I_bound / I_free, where I_free refers to the peak intensity in the absence of anle138b, and I_bound refers to the intensity at each titration point. Error bars indicate errors estimated from the signal-to-noise ratio of the NMR spectra. Source data are provided as a Source Data file.

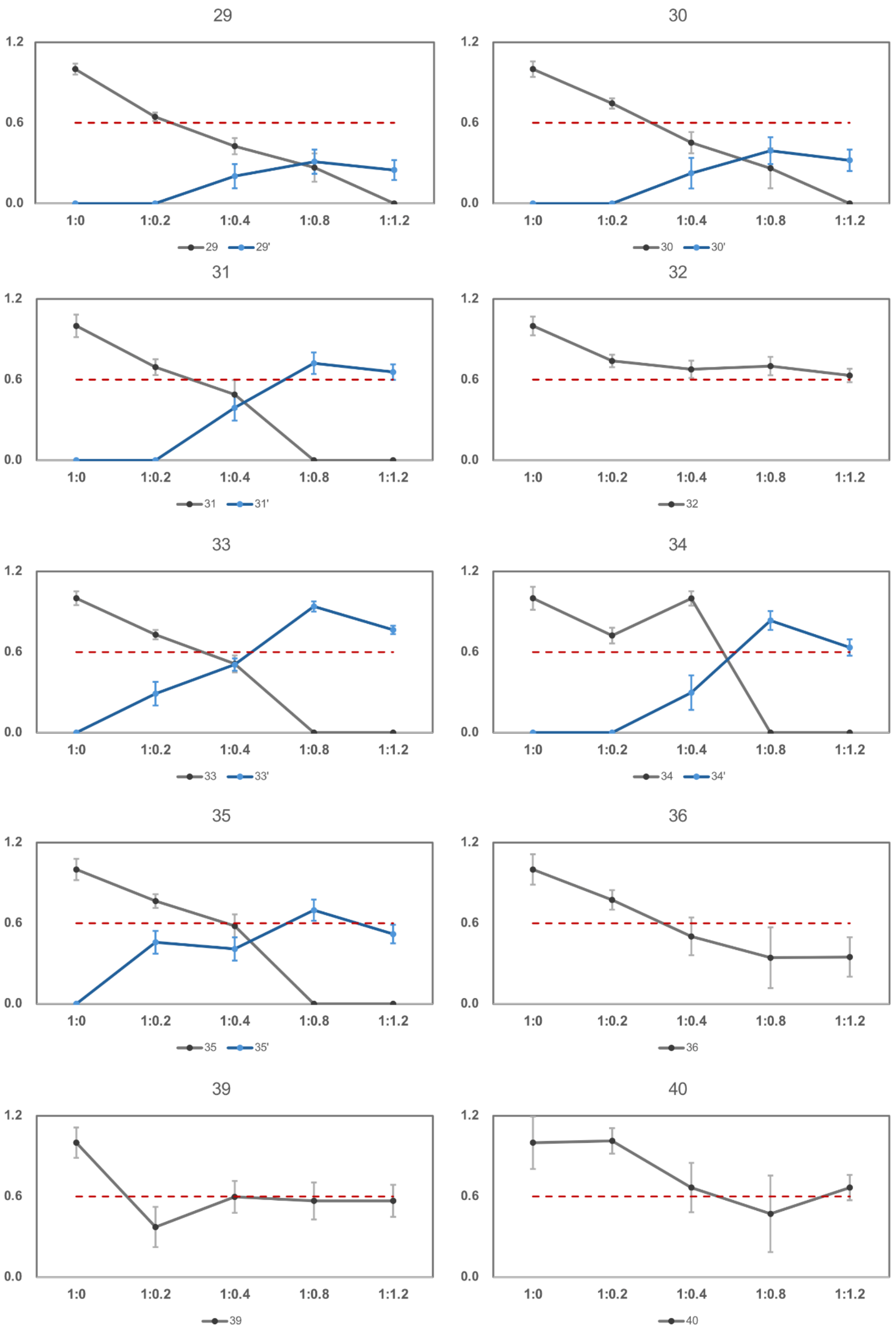
29
30
31
32
33
34
35
36
39
40
1.2
0.6
0.0
1:0
1:0.2
1:0.4
1:0.8
1:1.2
29
29'
30
30'
31
31'
32
33
33'
34
34'
35
35'
36
39
40

**Supplementary Figure 13 | Titration-dependent intensity changes indicating slow exchange binding of anle138b to residues 29–40 of L1 A$\beta_{40}$ fibrils.**

Signal intensity ratios (I_ratio) were monitored for selected residues of L1 A$\beta_{40}$ as a function of increasing anle138b molar ratios (anle138b: A$\beta_{40}$ molar ratio (SMPR) = 0 to 1.2) under post-treatment conditions. The gray curves represent normalized intensities of original peaks (I_free) observed at SMPR 0, and their changes upon ligand titration (I_bound). The blue curves indicate new peaks indicative of slow exchange. The dashed red line marks the 0.6 intensity threshold used as a reference for qualitative comparison. I_ratio = I_bound / I_free, where I_free refers to the peak intensity in the absence of anle138b, and I_bound refers to the intensity at each titration point. Error bars indicate errors estimated from the signal-to-noise ratio of the NMR spectra. Source data are provided as a Source Data file.

**a**

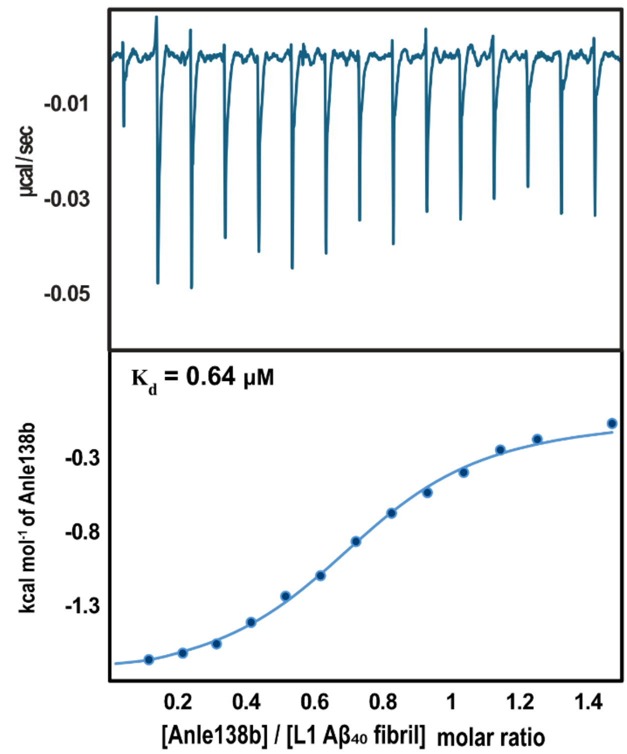

-0.01
-0.03
-0.05
µcal/sec
Kd = 0.64 µM
-0.3
-0.8
-1.3
kcal mol-1 of Anle138b
0.2
0.4
0.6
0.8
1
1.2
1.4
[Anle138b] / [L1 Aβ40 fibril] molar ratio


**b**

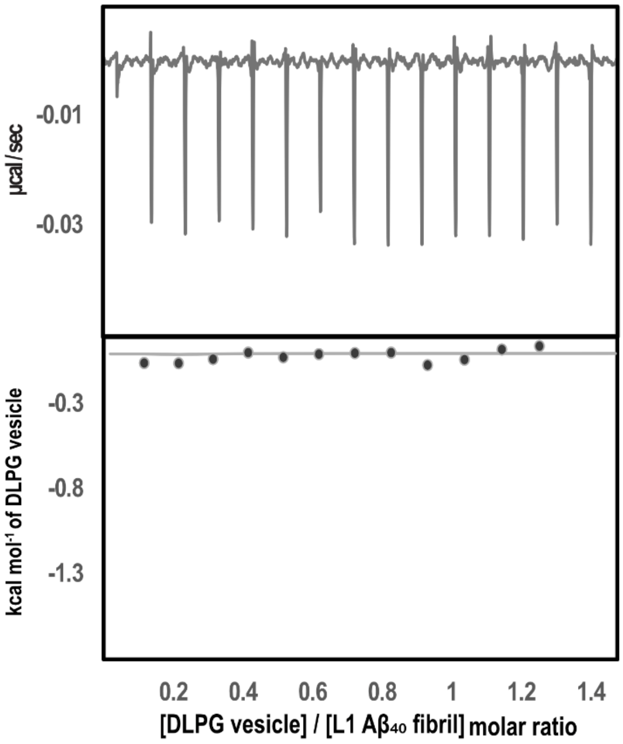

-0.01
-0.03
µcal/sec
-0.3
-0.8
-1.3
kcal mol-1 of DLPG vesicle
0.2
0.4
0.6
0.8
1
1.2
1.4
[DLPG vesicle] / [L1 Aβ40 fibril] molar ratio

**Supplementary Figure 14 | ITC analysis of anle138b and DLPG vesicle binding to L1 A$\beta_{40}$ fibrils.**

The thermodynamic parameters for binding interaction are summarized in Tab. 2.

**a.** Isothermal titration calorimetry (ITC) thermogram (top) and integrated binding isotherm (bottom) of anle138b (100 µM), formulated in DLPG vesicles (2 mM), titrated into L1 A$\beta_{40}$ fibrils (10 µM). The fitted curve yields a dissociation constant ($K_d$) of 0.64 µM and a binding stoichiometry of ~0.72 anle138b molecules per A$\beta_{40}$ monomer. **b.** Negative control: Titration of DLPG vesicles (2 mM) into L1 A$\beta_{40}$ fibrils (10 µM) produces negligible heat and a flat isotherm, indicating no detectable interaction between vesicles and fibrils. Source data are provided as a Source Data file.

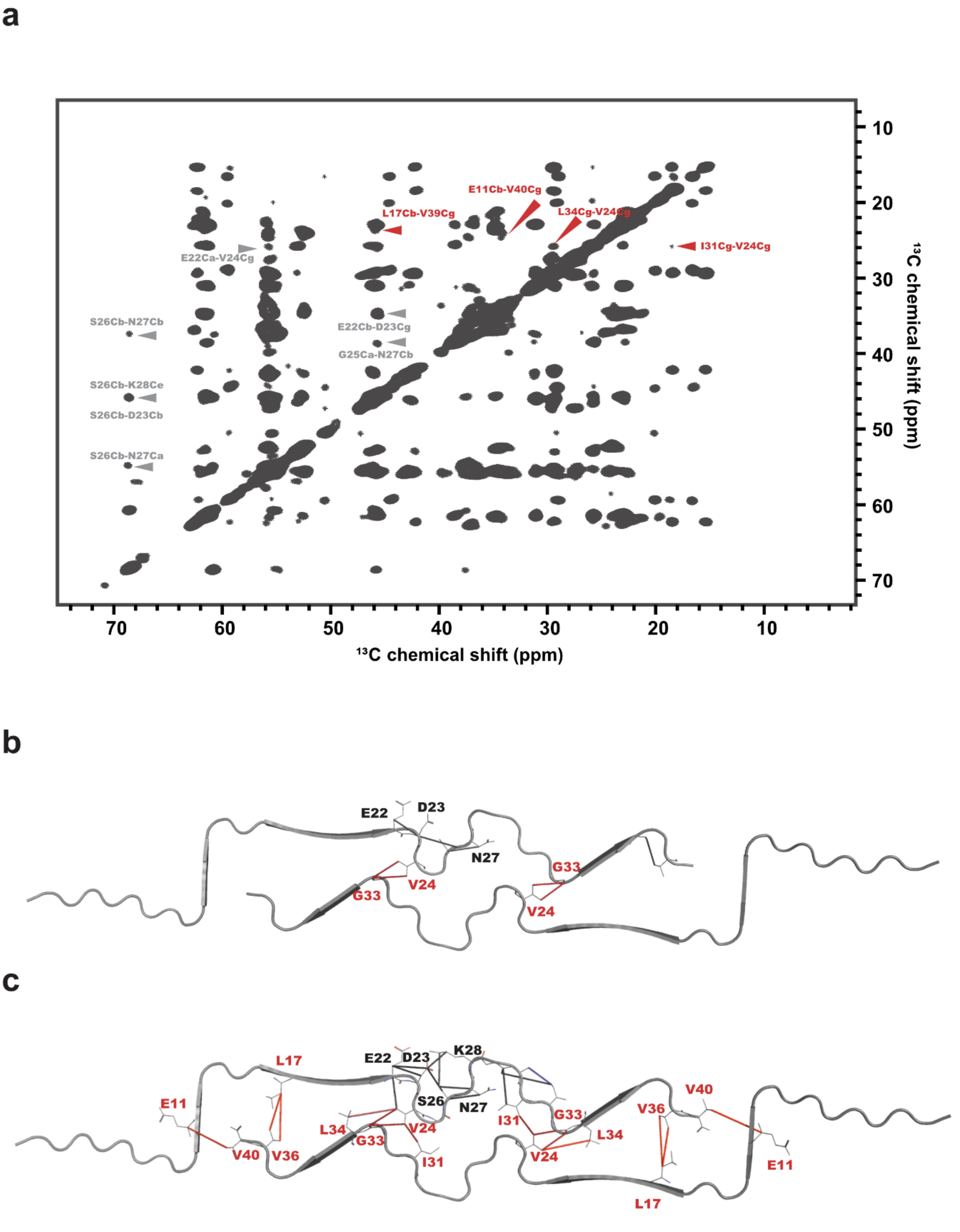
a
E11Cb-V40Cg
L17Cb-V39Cg
L34Cg-V24Cg
I31Cg-V24Cg
E22Ca-V24Cg
S26Cb-N27Cb
E22Cb-D23Cg
G25Ca-N27Cb
S26Cb-K28Ce
S26Cb-D23Cb
S26Cb-N27Ca
10
20
30
40
50
60
70
$^{13}$C chemical shift (ppm)
$^{13}$C chemical shift (ppm)
b
E22
D23
N27
G33
V24
G33
V24
c
E22
D23
K28
L17
E11
S26
N27
V40
V36
L34
G33
V24
I31
I31
G33
L34
V24
V36
V40
E11
L17

**Supplementary Figure 15 | 2D $^{13}C^{13}C$-DARR spectrum (265K, 850MHz) of L1 A$\beta_{40}$ fibrils.**

**a.** Aliphatic region of the 2D $^{13}C$–$^{13}C$ chemical shift correlation spectrum acquired at 265 K and 850 MHz (mixing time =200 ms). Gray arrows indicate medium-range intramolecular cross-peaks (2–4 Å); red arrows indicate medium-to-long-range intermolecular cross-peaks (3–5 Å). **b.** The structural model of L1 A$\beta_{40}$ fibrils shows the intramolecular contacts (gray bars, mixing time = 50 ms). **c.** The same structural model highlights the intra- and intermolecular contacts (gray and red bars, respectively, mixing time = 200 ms).

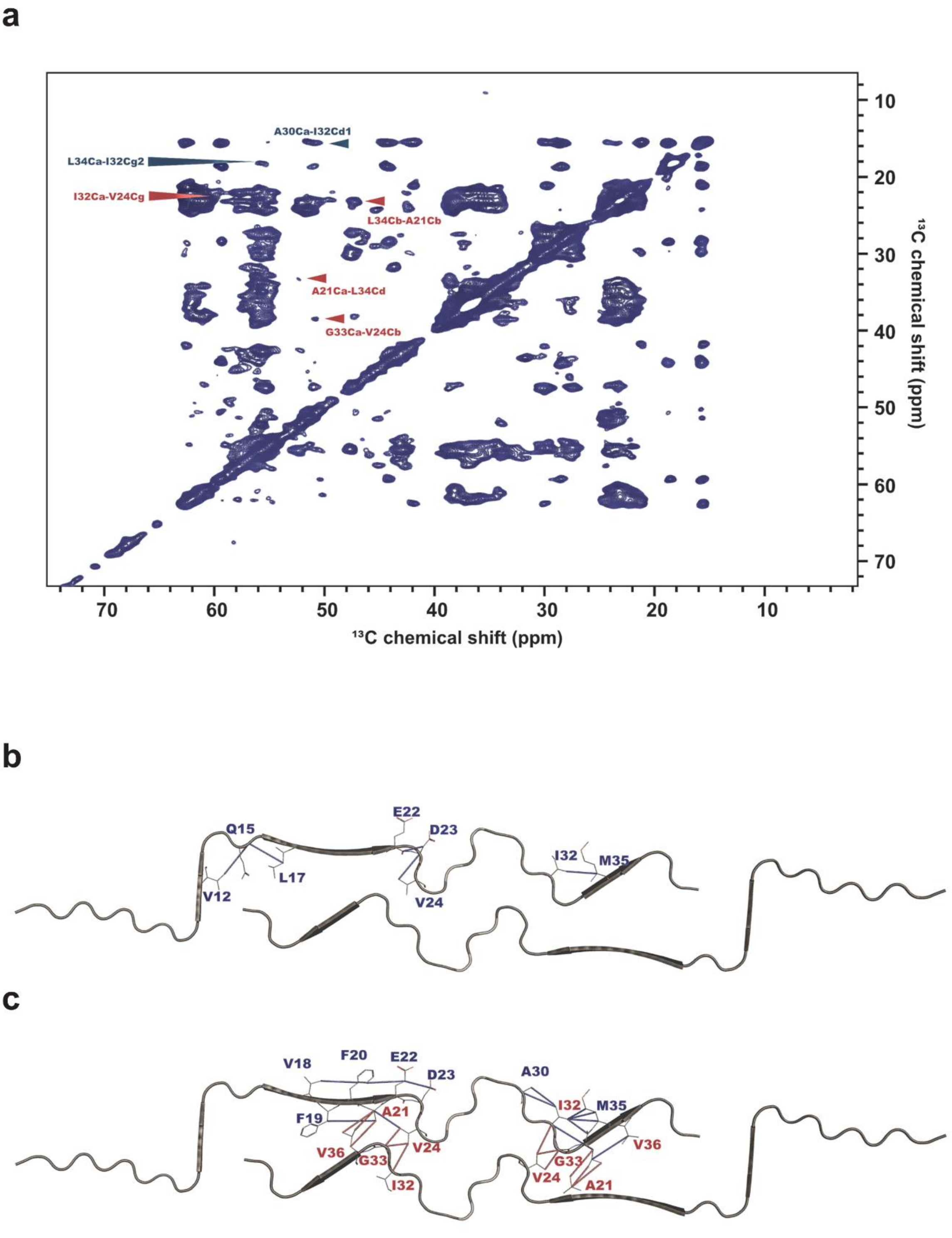
a
A30Ca-I32Cd1
L34Ca-I32Cg2
I32Ca-V24Cg
L34Cb-A21Cb
A21Ca-L34Cd
G33Ca-V24Cb
13C chemical shift (ppm)
13C chemical shift (ppm)
b
Q15
E22
D23
I32
M35
L17
V12
V24
c
V18
F20
E22
D23
A30
I32
M35
F19
A21
V36
G33
V24
I32
V36
G33
V24
A21

**Supplementary Figure 16 | 2D $^{13}C^{13}C$-DARR spectrum (265K, 850MHz) of L1 A$\beta_{40}$ fibrils (post-treatment condition).**

Anle138b was applied to L1 A$\beta_{40}$ fibrils after fibril formation (anle138b: A$\beta_{40}$ molar ratio (SMPR) = 1.2). **a.** Aliphatic region of the 2D $^{13}C$–$^{13}C$ chemical shift correlation spectrum acquired at 265 K and 850 MHz (mixing time =200 ms). Blue arrows indicate medium-range intramolecular cross-peaks (2–4 Å); red arrows indicate medium-to-long-range intermolecular cross-peaks (3–5 Å). **b.** The structural model of L1 A$\beta_{40}$ fibrils shows the intramolecular contacts (blue bars, mixing time = 50 ms). **c.** The same structural model highlights the intra- and intermolecular contacts (blue and red bars, respectively, mixing time = 200 ms).

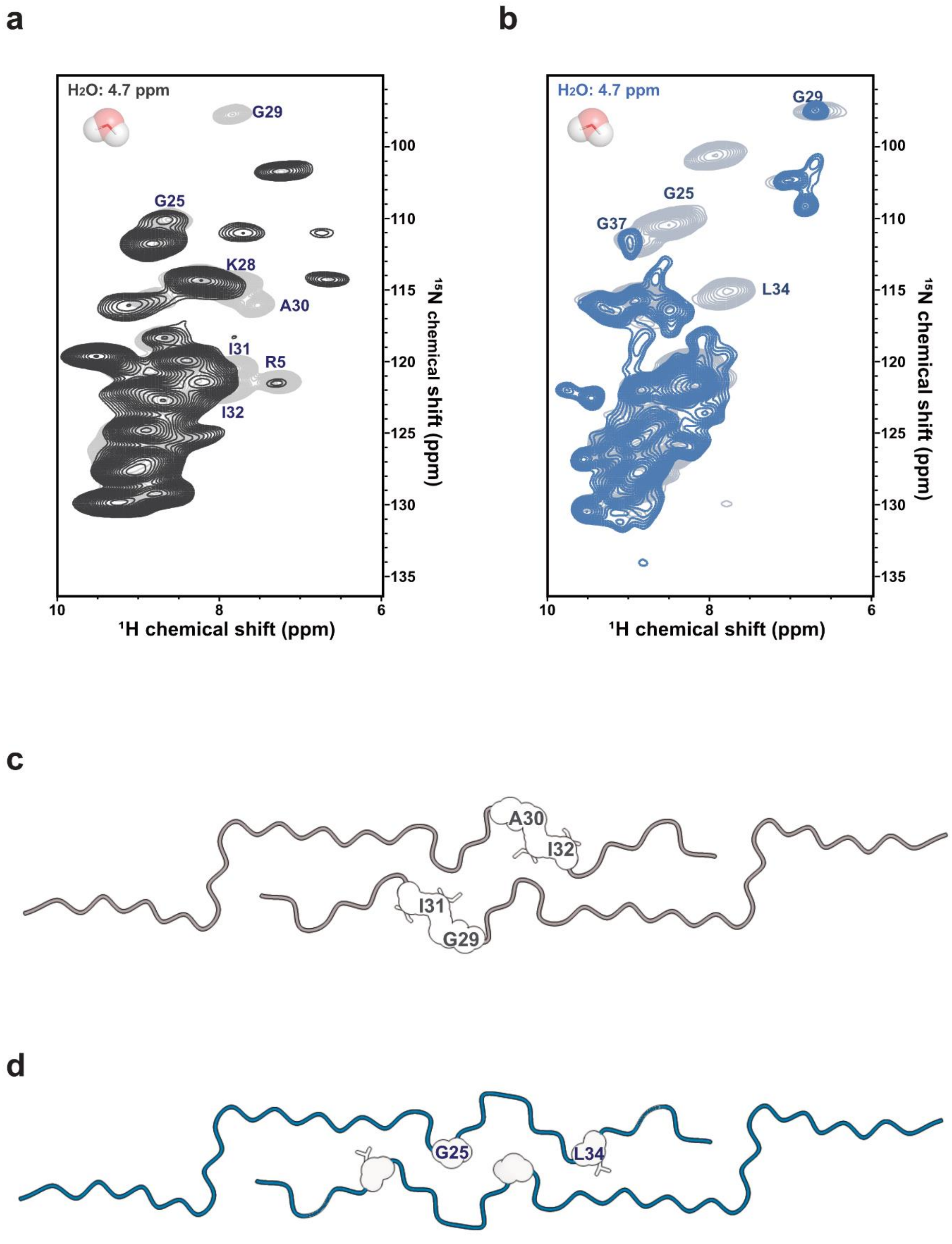

a
H2O: 4.7 ppm
G29
G25
K28
A30
I31
R5
I32
100
105
110
115
120
125
130
135
15N chemical shift (ppm)
10
8
6
1H chemical shift (ppm)
b
H2O: 4.7 ppm
G29
G25
G37
L34
c
A30
I32
I31
G29
d
G25
L34

**Supplementary Figure 17 | Comparison of $H_2O$-accessible amide groups in L1 Aβ$_{40}$ fibrils under control and post-treatment conditions.**

**a.** Light gray: 2D (H)NH spectrum of $^{2}H$, $^{13}C$, $^{15}N$-labeled L1 Aβ$_{40}$ fibrils (control). Dark gray: 2D plane at 4.7 ppm (water signal) in the H dimension was extracted from a 3D H(H)NH NOE spectrum with a 50 ms mixing time. Cross-peaks in dark gray indicate amide groups in close contact with $H_2O$ under control conditions. **b.** Light gray: 2D (H)NH spectrum of $^{2}H$, $^{13}C$, $^{15}N$-labeled L1 Aβ$_{40}$ fibrils under post-treatment conditions. Blue: 2D slice from a 3D H(H)NH NOE spectrum (50 ms mixing) at 4.7 ppm. Blue cross-peaks indicate water-accessible amides in the post-treatment condition. **c.** Structural map of control fibrils, with gray-colored residues indicating $H_2O$-accessible amides. **d.** Structural map of post-treatment fibrils, with blue-colored residues indicating water-accessible amides. Residues with no detectable water contact are shown as white spheres.

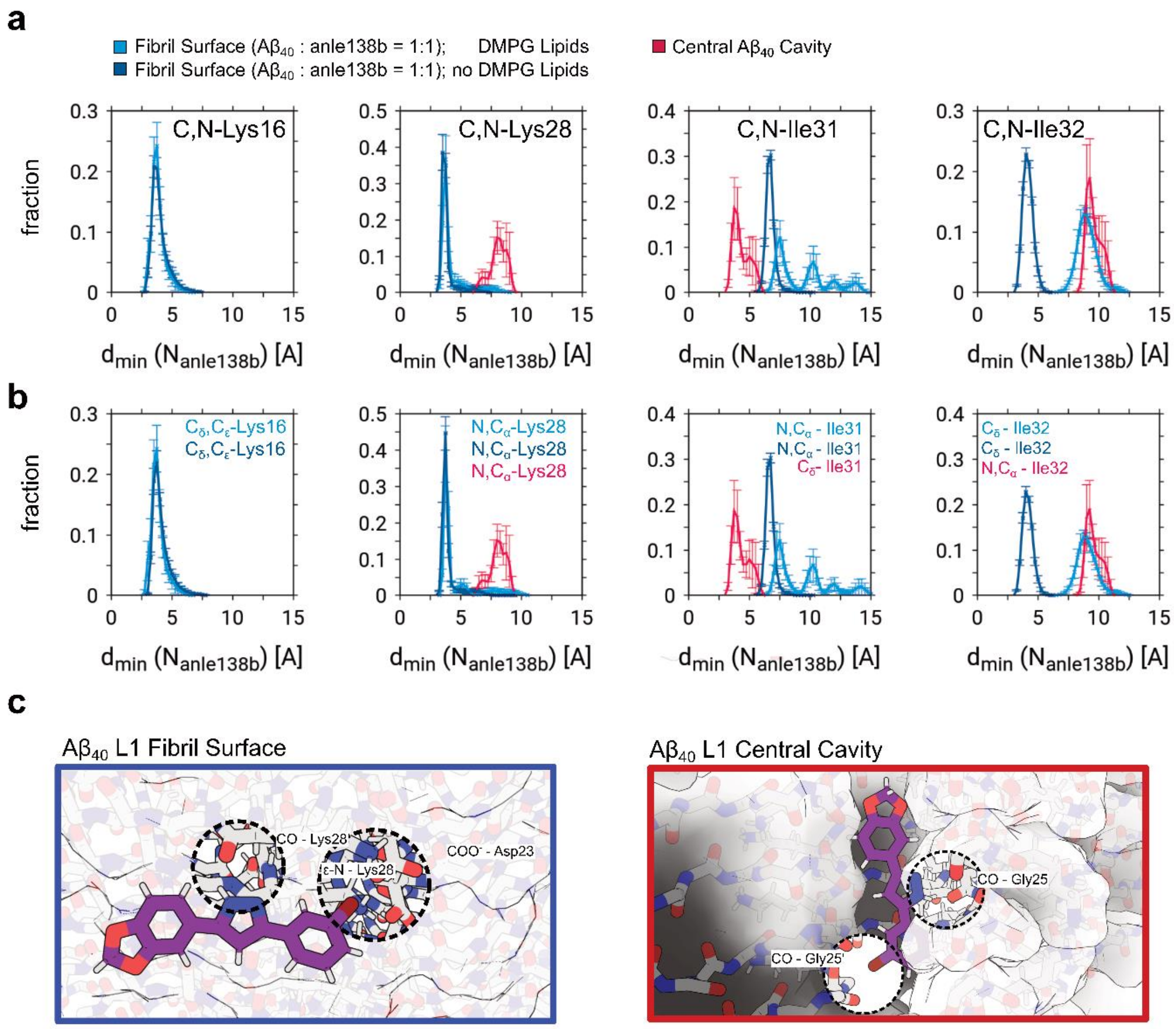
a
Fibril Surface (Aβ40 : anle138b = 1:1); DMPG Lipids
Fibril Surface (Aβ40 : anle138b = 1:1); no DMPG Lipids
Central Aβ40 Cavity
C,N-Lys16
C,N-Lys28
C,N-Ile31
C,N-Ile32
fraction
dmin (Nanle138b) [A]
b
Cδ,Cε-Lys16
N,Cα-Lys28
N,Cα - Ile31
Cδ- Ile31
Cδ - Ile32
N,Cα - Ile32
c
Aβ40 L1 Fibril Surface
CO - Lys28'
ε-N - Lys28
COO- - Asp23
Aβ40 L1 Central Cavity
CO - Gly25
CO - Gly25'

**Supplementary Figure 18 | Dominant binding poses and interatomic contacts of surface-bound and internally bound anle138b.**

**a.** Distributions of minimum distances derived from all N or C atoms of residues K16, K28, I31, and I32 to anle138b pyrazole nitrogen atoms for the simulation sets representing either surface-bound (blue and dark blue: without DMPG) or internally bound (red) anle138b. **b.** Distributions of minimum distances derived from the individual N and/or C atoms of residues K16, K28, I31, and I32 to anle138b pyrazole nitrogen atoms that best fit the distributions in **a** and **c.** For panels **a** and **b**, data are presented as mean ± SEM (indicated by error bars) for 10 replicates, respectively. Representative MD simulation snapshot of anle138b binding poses and interatomic contacts as observed for L1 A$\beta_{40}$ surface-bound (left panel) or internally bound (right panel). Circles highlight interatomic contacts: Lys28 carbonyl to anle138b pyrazole and Asp23/Lys28 side chain to anle138b bromophenyl interaction (left), as well as Gly25 carbonyl to anle138b pyrazole and Gly25 carbonyl (opposing protofilament) to anle138b bromophenyl interaction (right). DMPG lipids are not shown for clarity.

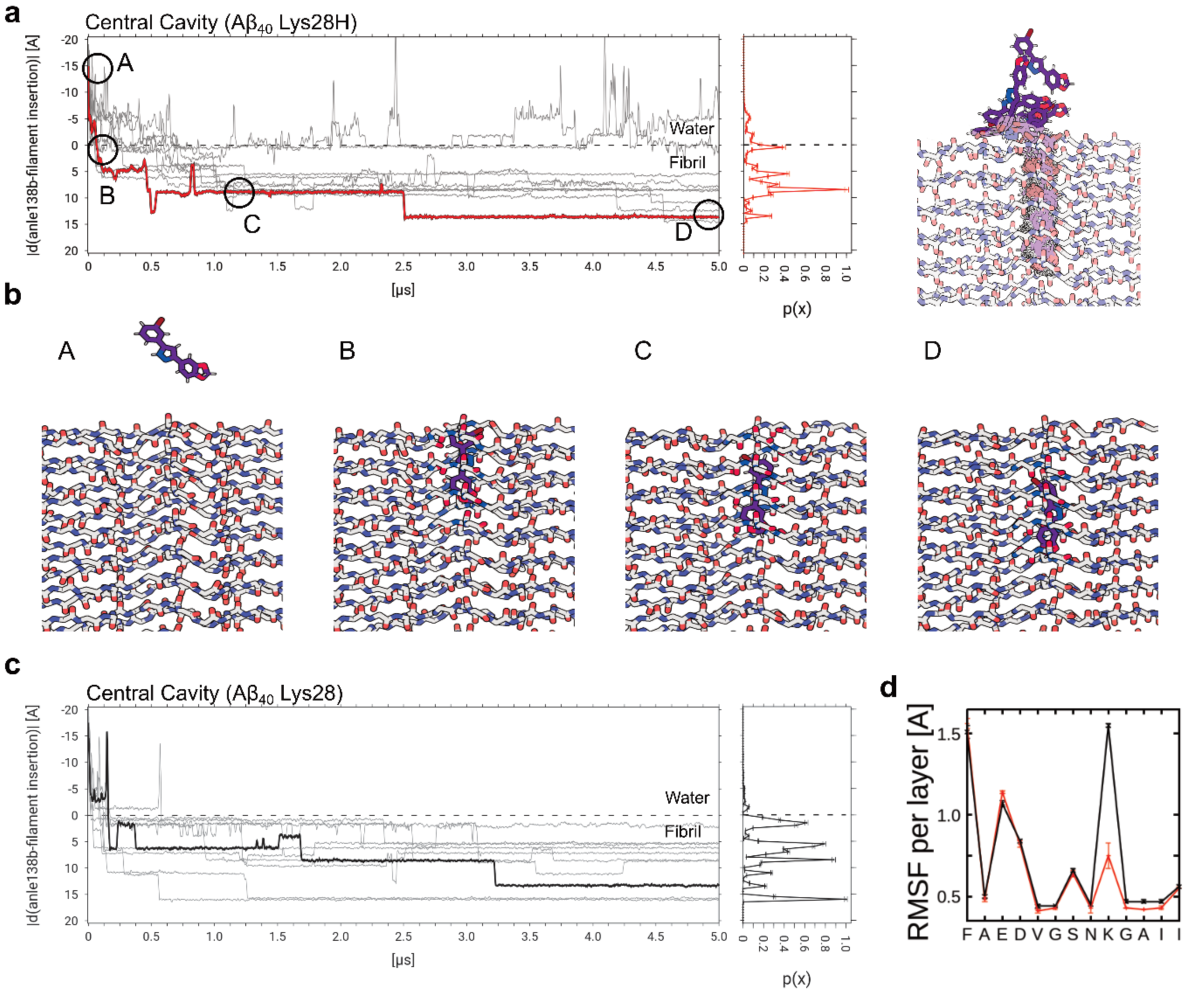

a
Central Cavity (Aβ40 Lys28H)
|d(anle138b-filament insertion)| [A]
Water
Fibril
A
B
C
D
[μs]
p(x)
b
A
B
C
D
c
Central Cavity (Aβ40 Lys28)
|d(anle138b-filament insertion)| [A]
Water
Fibril
[μs]
p(x)
d
RMSF per layer [A]
F A E D V G S N K G A I I

**Supplementary Figure 19 | Anle138b inserts spontaneously in the central cavity of A$\beta_{40}$ L1 fibrils.**

**a.** Distance of the anle138b pyrazole nitrogen atoms to the fibril center-of-mass (representative trajectory as bold lines: color red; others as shaded thin lines for clarity: color gray) and **b.** snapshots (A, B, C, D) from MD simulations of anle138b spontaneously binding internally to the cavity of L1, as well as it's discrete translational motion along the fibril axis. **c.** Distance of the anle138b pyrazole nitrogen atoms to the fibril center-of-mass for simulations with deprotonated Lys28 i.e. disrupted hydrogen bond interaction between Asp23 and Lys28. The **histograms** (panels on the right) in **a** and **c** were calculated over the full trajectory length, respectively. Simulations in **a** show that deeper or comparable insertion to **c** does not occur on the multi μs-time scales accessible by the reported MD simulations. **d.** Average root mean squared fluctuations (RMSF) for residues of the loop region with (red) and without (black) intact Asp23-Lys28 hydrogen bond. Data are presented as mean values ± SEM (depicted by error bars). Source data are provided as a Source Data file.

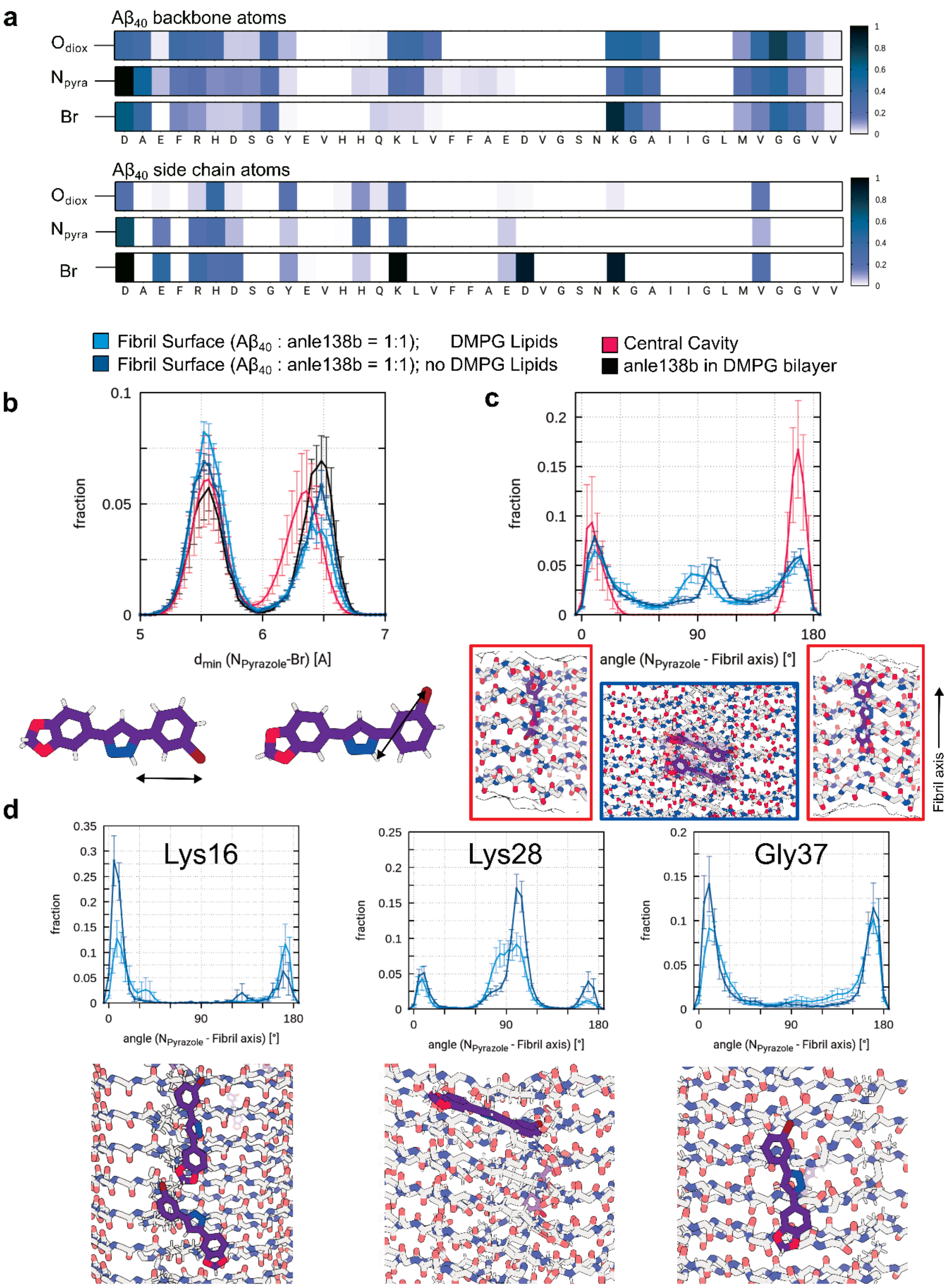

a
Aβ40 backbone atoms
Odiox
Npyra
Br
D A E F R H D S G Y E V H H Q K L V F F A E D V G S N K G A I I G L M V G G V V
Aβ40 side chain atoms
Odiox
Npyra
Br
D A E F R H D S G Y E V H H Q K L V F F A E D V G S N K G A I I G L M V G G V V
Fibril Surface (Aβ40 : anle138b = 1:1); DMPG Lipids
Fibril Surface (Aβ40 : anle138b = 1:1); no DMPG Lipids
Central Cavity
anle138b in DMPG bilayer
b
fraction
dmin (NPyrazole-Br) [A]
c
fraction
angle (NPyrazole - Fibril axis) [°]
Fibril axis
d
Lys16
Lys28
Gly37
fraction
angle (NPyrazole - Fibril axis) [°]

**Supplementary Figure 20 | Anle138b binding modes to the surface and central cavity of L1 A$\beta_{40}$ fibrils.**

**a.** Heat maps report polar contacts between backbone and side chain atoms of individual L1 A$\beta_{40}$ fibril structure residues and polar moieties of anle138b, respectively. Scale bars indicate contact probabilities. **b.** Distance distribution of anle138b pyrazole N to Bromine atom (blue – surface-bound anle138b; red - internal binding pose, black – anle138b in DMPG bilayer). Data are presented as mean ± s.e.m. (indicated by error bars) for 10 replicates, respectively. **c.** Anle138b alignment with respect to the fibril axis when bound either internally to the loop region (red) or to the surface of the L1 A$\beta_{40}$ fibrils (blue). Data are presented as mean ± s.e.m. (indicated by error bars) for 10 replicates, respectively. **d.** Anle138b alignment with respect to the fibril axis when bound to residues Lys16, Lys28, and Gly37 of the L1 A$\beta_{40}$ fibril surface. Data are presented as mean ± s.e.m. (indicated by error bars) for 10 replicates, respectively. Representative snapshots of anle138b binding poses are shown. DMPG lipids are not depicted for clarity. Source data are provided as a Source Data file.

a

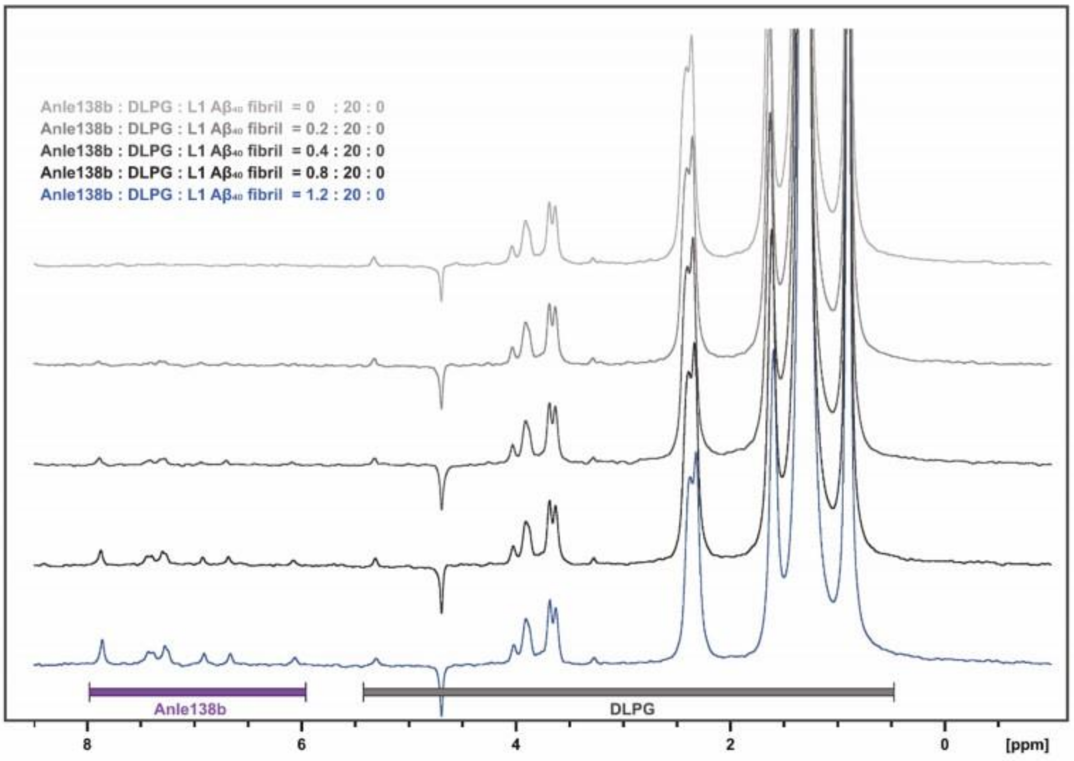

Anle138b : DLPG : L1 Aβ40 fibril = 0 : 20 : 0
Anle138b : DLPG : L1 Aβ40 fibril = 0.2 : 20 : 0
Anle138b : DLPG : L1 Aβ40 fibril = 0.4 : 20 : 0
Anle138b : DLPG : L1 Aβ40 fibril = 0.8 : 20 : 0
Anle138b : DLPG : L1 Aβ40 fibril = 1.2 : 20 : 0
Anle138b
DLPG
8
6
4
2
0
[ppm]


b

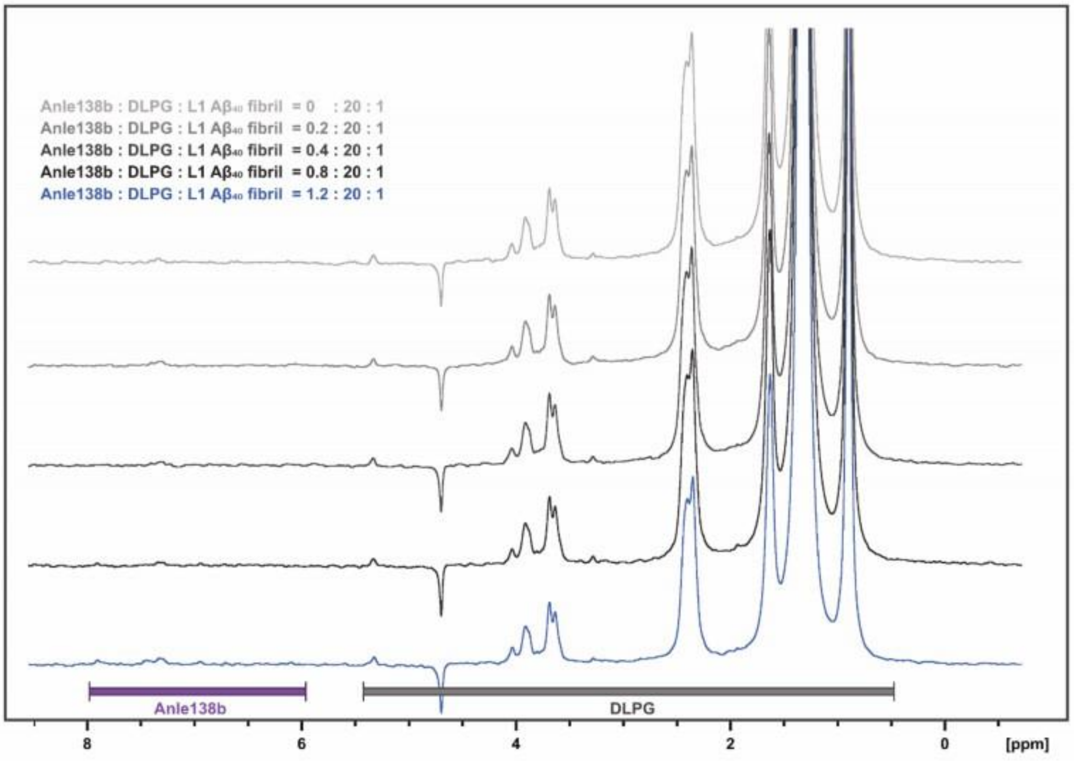

Anle138b : DLPG : L1 Aβ40 fibril = 0 : 20 : 1
Anle138b : DLPG : L1 Aβ40 fibril = 0.2 : 20 : 1
Anle138b : DLPG : L1 Aβ40 fibril = 0.4 : 20 : 1
Anle138b : DLPG : L1 Aβ40 fibril = 0.8 : 20 : 1
Anle138b : DLPG : L1 Aβ40 fibril = 1.2 : 20 : 1
Anle138b
DLPG
8
6
4
2
0
[ppm]

**Supplementary Figure 21 | 1D $^1$H NMR spectra showing anle138b incorporation into DLPG vesicles and its binding to L1 A$\beta_{40}$ fibrils under post-treatment conditions.**

**a.** 1D $^1$H NMR spectra (ns=128) showing increasing concentrations of anle138b incorporated into DLPG vesicles. The purple region indicates anle138b signals, and the gray region indicates DLPG signals. **b.** 1D $^1$H NMR spectra (ns=128) of the supernatant obtained after ultracentrifugation of L1 A$\beta_{40}$ fibrils treated under the conditions shown in panel a (after 1h 37°C. incubation). The decrease in free anle138b signals with increasing concentration indicates that most anle138b is bound to the fibrils.

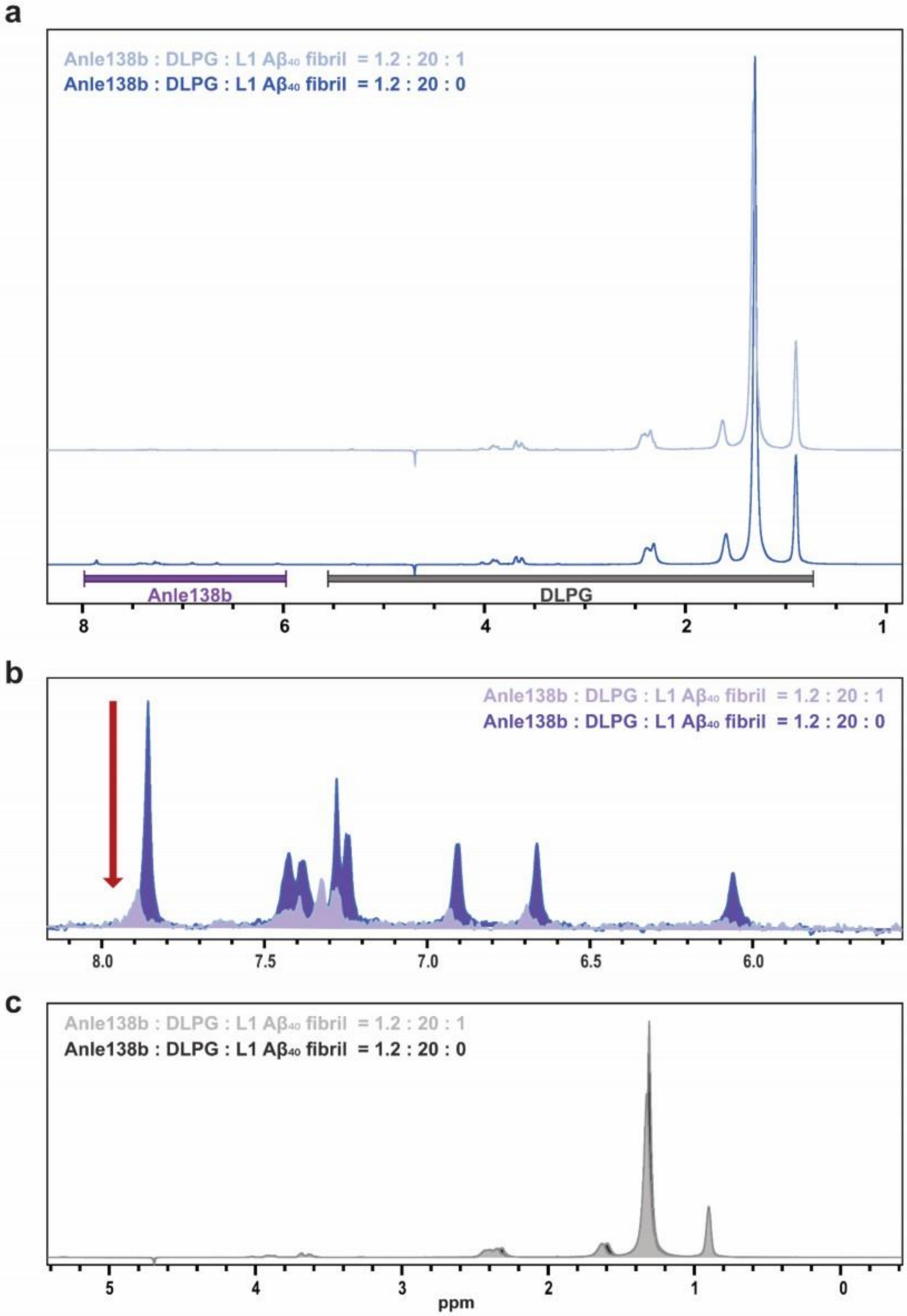

a
Anle138b : DLPG : L1 Aβ40 fibril = 1.2 : 20 : 1
Anle138b : DLPG : L1 Aβ40 fibril = 1.2 : 20 : 0
Anle138b
DLPG
8
6
4
2
1
b
Anle138b : DLPG : L1 Aβ40 fibril = 1.2 : 20 : 1
Anle138b : DLPG : L1 Aβ40 fibril = 1.2 : 20 : 0
8.0
7.5
7.0
6.5
6.0
c
Anle138b : DLPG : L1 Aβ40 fibril = 1.2 : 20 : 1
Anle138b : DLPG : L1 Aβ40 fibril = 1.2 : 20 : 0
5
4
3
2
1
0
ppm

**Supplementary Figure 22 | $^{1}$H NMR spectra showing the decrease of free anle138b in the supernatant after incubation with** L1 **A$\beta_{40}$ fibrils.**

**a.** Overlaid $^{1}$H NMR spectra (ns=1024) of samples without A$\beta_{40}$ fibrils (dark blue) and with fibrils (light blue). The fibril-containing sample was incubated with anle138b and A$\beta_{40}$ fibrils at a 1.2:1 molar ratio for 1 hour, followed by ultracentrifugation, and only the supernatant was analyzed. **b.** In the δ 8.0–6.0 ppm region, the anle138b peaks highlighted in purple showed a significant decrease after fibril treatment (red arrow). **c.** In the δ 5.0–0.5 ppm region, the DLPG peaks highlighted in gray remained nearly unchanged under both conditions, indicating a consistent amount of vesicles and serving as a reference for quantification. Quantitative analysis revealed approximately a 70% decrease in the integrated anle138b peak, with the signal-to-noise ratio (S/N) dropping from 52.1 to 11.0. This suggests that a substantial amount of anle138b was bound to or removed by the fibrils, resulting in its depletion from the supernatant.

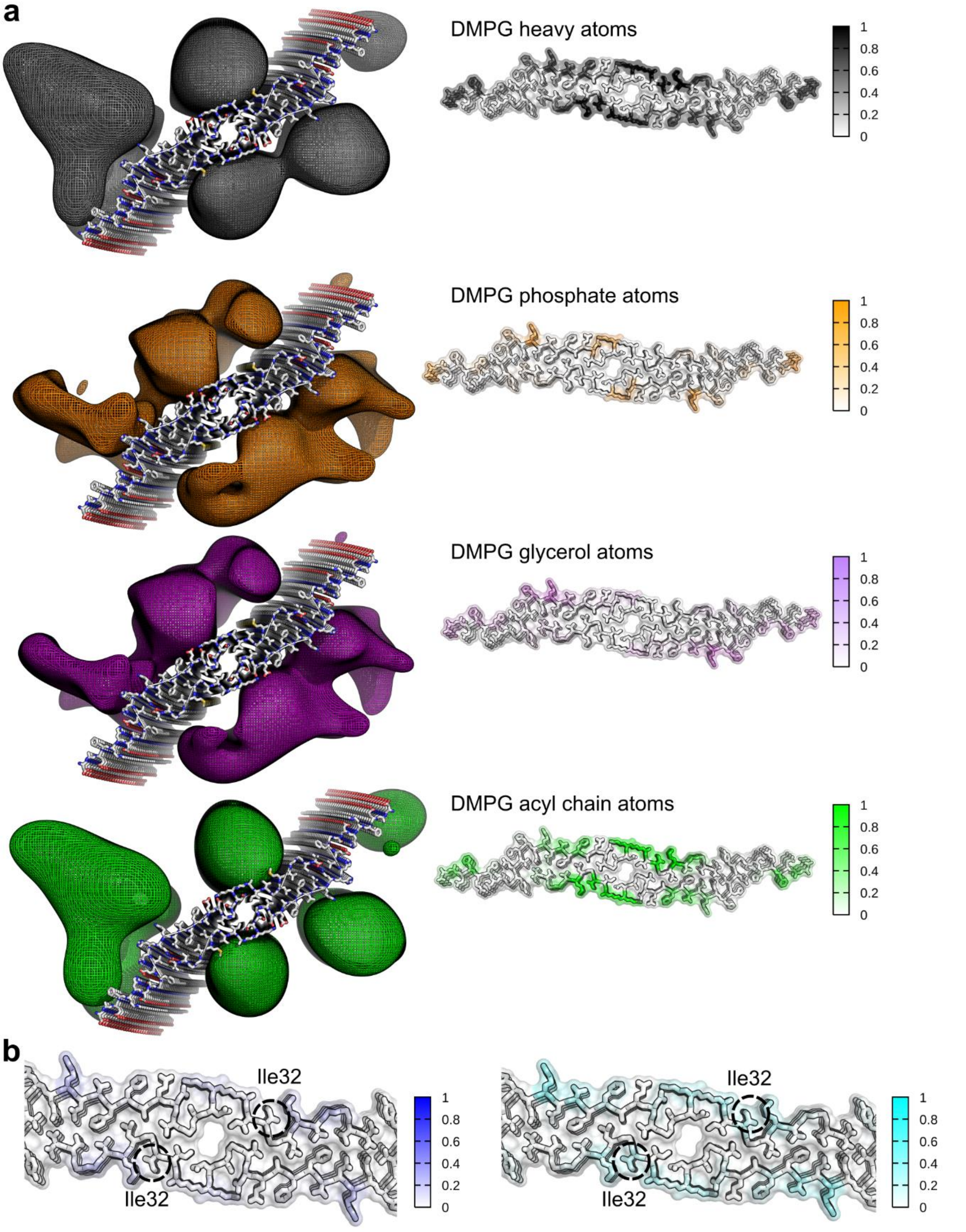


**Supplementary Figure 23 | Determination of the binding region on L1 A$\beta_{40}$ fibrils for lipids.**

(**a**, left) 3D probability density of the DMPG lipid atomic positions and (**a**, right) atomic models with residues colored according to their interaction frequencies with all lipid heavy atoms

(black), the phosphate group (orange), glycerol group (purple), the acyl chains (green) throughout the MD simulations for the L1 A$\beta_{40}$ fibril structure. Density maps were visualized as isomesh and contoured at 2σ. (**b**) Interaction frequencies of anle138b nitrogen atoms with residues of the L1 A$\beta_{40}$ fibril structure (left – in the presence of DMPG, right – in the absence of DMPG). The location of residue I32 is highlighted by a black circle. Residues are colored with the color saturation linearly increasing with interaction frequencies between 0 and 100%. Source data are provided as a Source Data file.

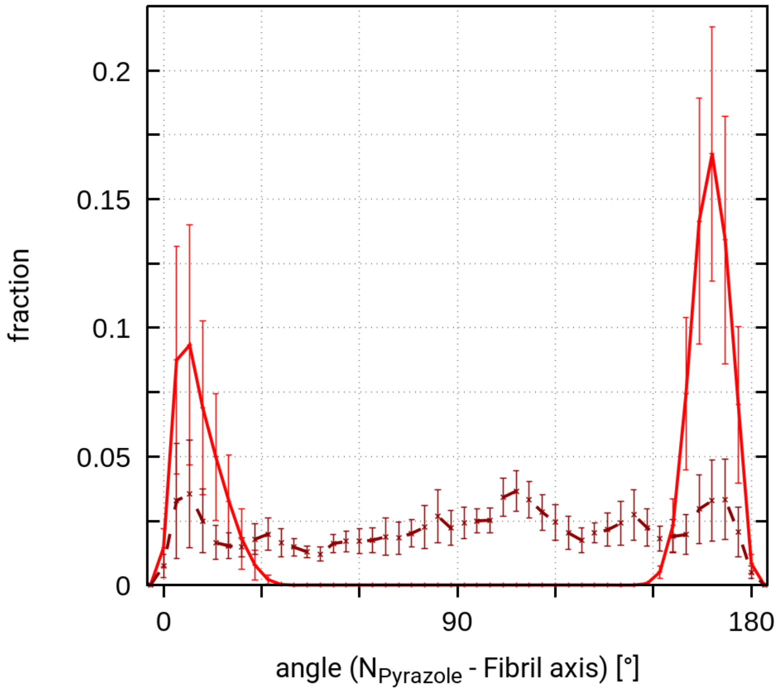


**Supplementary Figure 24 | Anle138b orientations in MD simulations of truncated fibril models with flat-bottomed potentials around the central cavity of L1 A$\beta_{40}$ fibrils.**

Anle138b alignment with respect to the fibril axis and when bound either internally to the central loop region (red solid line, as shown in Fig. S19) or unbound during the MD simulations of truncated fibril models (dark-red broken line). Data are presented as mean ± s.e.m. (indicated by error bars). Source data are provided as a Source Data file.

**Supplementary Table 1 | Experimental parameters of NMR data acquisition on the sample.**

| Exp | Spectrometer | MAS (kHz) rotor | Transfer | Nucleus | time cp (ms) | rf (kHz) | Ramp | Temperature | Sample |
|---|---|---|---|---|---|---|---|---|---|
| 3D (H)CANH | 800, Advance III | 55 (1.3mm) | $^{1}$H-$^{13}$C-CP | $^{1}$H | 6.5 | 96 | 80-100% | 235K | $^{2}$H,$^{13}$C,$^{15}$N $A\beta_{40}$ fibril<br>$^{2}$H,$^{13}$C,$^{15}$N $A\beta_{40}$ fibril: Anle138b = 1:0.2, 1: 0.4, 1:0.8, 1:1.2 (titration) |
| | | | | $^{13}$C | | 88 | square | | |
| | | | $^{13}$C-$^{15}$N-CP | $^{13}$C | 20 | 139.1 | tangent | | |
| | | | | $^{15}$N | | 57 | square | | |
| | | | $^{15}$N-$^{1}$H-CP | $^{1}$H | | 145 | 100-80% | | |
| | | | | $^{15}$N | 0.7 | 85 | | | |
| 3D (H)coCAcoNH | 800, Advance III | 55 (1.3mm) | $^{1}$H-$^{13}$C-CP | $^{1}$H | 2.9 | 22 | 85-100% | 235K | $^{2}$H,$^{13}$C,$^{15}$N $A\beta_{40}$ fibril: Anle138b = 1:1.2 |
| | | | | $^{13}$C | | 17 | square | | |
| | | | $^{13}$C-$^{15}$N-CP | $^{13}$C | 25 | 17 | tangent | | |
| | | | | $^{15}$N | | 15 | square | | |
| | | | $^{15}$N-$^{1}$H-CP | $^{1}$H | 8 | 24 | 100-80% | | |
| | | | | $^{15}$N | 0.85 | 20.7 | | | |
| 2D (H)NH | 800, Advance III | 55 (1.3mm) | $^{15}$N-$^{1}$H-CP | $^{1}$H | 3.1 | 97 | 100-80% | 235K | $^{2}$H,$^{13}$C,$^{15}$N $A\beta_{40}$ fibril: Anle138b = 1:1.2 |
| | | | | $^{15}$N | | 170 | | | |
| | | | $^{1}$H-$^{15}$N-CP | $^{1}$H | 0.9 | 104 | 80-100% | | |
| | | | | $^{15}$N | | 170 | | | |
| 3D H(H)NH NOE | 800, Advance III | 55 (1.3mm) | $^{15}$N-$^{1}$H-CP | $^{1}$H | 3.1 | 90 | 100-80% | 235K | $^{2}$H,$^{13}$C,$^{15}$N $A\beta_{40}$ fibril: Anle138b = 1:1.2 |
| | | | | $^{15}$N | | 152 | | | |
| | | | $^{1}$H-$^{15}$N-CP | $^{1}$H | 0.9 | 99 | 80-100% | | |

| | | | | | | | | | |
|---|---|---|---|---|---|---|---|---|---|
| | | | | $^{15}$N | | 166 | | | |
| 2D $^{13}$C-$^{13}$C-DARR (20ms, 50ms, 200ms, 400ms) | 850, NEO | 17 (3.2mm) | $^{1}$H-$^{13}$C-CP | $^{1}$H | | 80 | 100-80% | 265K | $^{1}$H,$^{13}$C,$^{15}$N A$\beta_{40}$ fibril<br><br>$^{1}$H,$^{13}$C,$^{15}$N A$\beta_{40}$ fibril: Anle138b = 1:1.2 (Post-treatment condition)<br><br>$^{1}$H,$^{13}$C,$^{15}$N A$\beta_{40}$ fibril: Anle138b = 1:1.2 (Pre-treatment condition) |
| | | | | $^{13}$C | 1.1 | 88 | | | |
| 3D (H)CANH | 850, NEO | 55 (1.3mm) | $^{1}$H-$^{13}$C-CP | $^{1}$H | 2.9 | 22 | 85-100% | 235K | $^{1}$H,$^{13}$C,$^{15}$N A$\beta_{40}$ fibril |
| | | | | $^{13}$C | | 17 | square | | |
| | | | $^{13}$C-$^{15}$N-CP | $^{13}$C | 25 | 17 | tangent | | |
| | | | | $^{15}$N | | 15 | square | | |
| | | | $^{15}$N-$^{1}$H-CP | $^{1}$H | | 24 | 100-80% | | |
| | | | | $^{15}$N | 0.8 | 20.7 | | | |
| 2D (H)NCA | 850, NEO | 17 (3.2mm) | $^{13}$C-$^{15}$N-CP | $^{13}$C | 3 | 16 | 100-90% | 265K | $^{1}$H,$^{13}$C,$^{15}$N A$\beta_{40}$ fibril<br><br>$^{1}$H,$^{13}$C,$^{15}$N A$\beta_{40}$ fibril: Anle138b = 1:1.2 (Pre-treatment condition)<br><br>$^{1}$H,$^{13}$C,$^{15}$N A$\beta_{40}$ fibril: Anle138b = 1:1.2 (Pre-treatment condition) |
| | | | | $^{15}$N | | 18 | | | |
| | | | $^{1}$H-$^{15}$N-CP | $^{1}$H | 1.5 | 83 | 80-100% | | |
| | | | | $^{15}$N | | 73 | | | |

| | | | | | | | | | |
|---|---|---|---|---|---|---|---|---|---|
| 2D $^{13}C$-$^{13}C$-DARR (20ms) | 850, NEO | 17 (3.2mm) | $^{1}H$-$^{13}C$-CP | $^{1}H$ | | 80 | 100-80% | 265K | ILE $^{13}C$,$^{15}N$ $A\beta_{40}$ fibril<br>ILE $^{13}C$,$^{15}N$ $A\beta_{40}$ fibril: Anle138b = 1:1.2 (Post-treatment condition)<br>ILE $^{13}C$,$^{15}N$ $A\beta_{40}$ fibril: Anle138b = 1:1.2 (Pre-treatment condition) |
| | | | | $^{13}C$ | 1.5 | 88 | | | |
| 2D $^{13}C$-$^{13}C$-DARR (20ms) | 850, NEO | 17 (3.2mm) | $^{1}H$-$^{13}C$-CP | $^{1}H$ | | 80 | 100-80% | 265K | LYS $^{13}C$,$^{15}N$ $A\beta_{40}$ fibril<br>LYS $^{13}C$,$^{15}N$ $A\beta_{40}$ fibril: Anle138b = 1:1.2 (Post -fibril condition)<br>LYS $^{13}C$,$^{15}N$ $A\beta_{40}$ fibril: Anle138b = 1:1.2 (Pre-treatment condition) |
| | | | | $^{13}C$ | 1.2 | 88 | | | |
| 2D $^{13}C$-$^{13}C$-RFDR (2.6ms) | 600, Advance III<br>e = 6 ~8<br>TEMTRIPol-1 | 10 (3.2mm) | $^{1}H$-$^{13}C$-CP | $^{1}H$ | | 98 | 90-100% | 100K (DNP) | ILE $^{13}C$,$^{15}N$ $A\beta_{40}$ fibril: Anle138b = 1:1.2 (Post-treatment condition)<br>ILE $^{13}C$,$^{15}N$ $A\beta_{40}$ fibril: Anle138b = 1:1.2 (Pre-treatment condition) |
| | | | | $^{13}C$ | 0.9 | 77 | | | |
| 2D $^{13}C$-$^{13}C$-DARR (50ms) | 600, Advance III<br>e = 6 ~8<br>TEMTRIPol-1 | 10 (3.2mm) | $^{1}H$-$^{13}C$-CP | $^{1}H$ | | 70.8 | 90-100% | 100K (DNP) | LYS $^{13}C$,$^{15}N$ $A\beta_{40}$ fibril: Anle138b = 1:1.2 (Post-treatment condition) |

| | | | | | | | | | |
|---|---|---|---|---|---|---|---|---|---|
| | | | | $^{13}$C | 0.6 | | | | LYS $^{13}$C,$^{15}$N A$\beta_{40}$ fibril:<br>Anle138b = 1:1.2<br>(Pre-treatment condition) |
| 2D NHHC | 600, Advance III<br>e = 6 ~8<br>TEMTRIPol-1 | 10 (3.2mm) | $^{1}$H-$^{15}$N-CP | $^{1}$H | 0.35 | 49 | 90-100% | 100K (DNP) | $^{1}$H,$^{13}$C,$^{15}$N A$\beta_{40}$ fibril:<br>Anle138b = 1:1.2<br>(Pre-treatment condition)<br><br>$^{1}$H,$^{13}$C,$^{15}$N A$\beta_{40}$ fibril:<br>Anle138b = 1:1.2<br>(Pre-treatment condition) |
| | | | | $^{15}$N | | 32 | | | |
| | | | $^{15}$N-$^{1}$H--CP | $^{15}$N | | 32 | | | |
| | | | | $^{1}$H | 0.35 | 49 | 80-100% | | |
| | | | $^{1}$H-$^{13}$C-CP | $^{1}$H | | 61 | 80-100% | | |
| | | | | $^{13}$C | 0.6 | 84 | | | |
| 2D NHHC | 600, Advance III<br>e = 6 ~8<br>TEMTRIPol-1 | 10 (3.2mm) | $^{1}$H-$^{15}$N-CP | $^{1}$H | 0.36 | 39 | 90-100% | 100K (DNP) | LYS $^{13}$C,$^{15}$N A$\beta_{40}$ fibril:<br>Anle138b = 1:1.2<br>(Post-treatment condition)<br><br>LYS $^{13}$C,$^{15}$N A$\beta_{40}$ fibril:<br>Anle138b = 1:1.2<br>(Pre-treatment condition) |
| | | | | $^{15}$N | | 31 | | | |
| | | | $^{15}$N-$^{1}$H--CP | $^{15}$N | | 31 | | | |
| | | | | $^{1}$H | 0.6 | 39 | 80-100% | | |
| | | | $^{1}$H-$^{13}$C-CP | $^{1}$H | | 67 | 80-100% | | |
| | | | | $^{13}$C | 0.7 | 61 | | | |
| 2D NHHC | 600, Advance III<br>e = 6 ~8<br>TEMTRIPol-1 | 10 (3.2mm) | $^{1}$H-$^{15}$N-CP | $^{1}$H | 0.5 | 43 | 90-100% | 100K (DNP) | ILE $^{13}$C,$^{15}$N A$\beta_{40}$ fibril:<br>Anle138b = 1:1.2<br>(Post-treatment condition)<br><br>ILE $^{13}$C,$^{15}$N A$\beta_{40}$ fibril:<br>Anle138b = 1:1.2<br>(Pre-treatment fibril condition) |
| | | | | $^{15}$N | | 33 | | | |
| | | | $^{15}$N-$^{1}$H--CP | $^{15}$N | | 33 | | | |
| | | | | $^{1}$H | 0.3 | 43 | 80-100% | | |
| | | | $^{1}$H-$^{13}$C-CP | $^{1}$H | | 98 | 80-100% | | |
| | | | | $^{13}$C | 0.7 | 77 | | | |

**Supplementary Table 2 | Thermodynamic parameters obtained from ITC titrations of anle138b (in DLPG vesicle) or DLPG vesicle alone into L1 $A\beta_{40}$ fibrils.**

Control titration results using DLPG vesicles without anle138b are shown in Supplementary Fig. 14 b, while titrations with DLPG vesicles containing anle138b are shown in Fig. 4 b and Supplementary Fig. 14a.

| | **[Anle138b (DLPG vesicle)] / [L1 $A\beta_{40}$ fibril]** | **[DLPG vesicle] / [L1 $A\beta_{40}$ fibril]** |
|---|---|---|
| **[Cell] (μM)** | 10.00 | 10.00 |
| **[Syringe] (μM)** | 100.00 | 100.00 |
| **N (sites)** | 0.72 ± 0.01 | |
| **KD (μM)** | 0.64 ± 0.068 | |
| **ΔH (kcal/mol)** | -1.84 ± 0.039 | |
| **ΔG (kcal/mol)** | -8.45 | |
| **-TΔS (kcal/mol)** | -6.62 | |

**Supplementary Table 3 | Cryo-EM structure determination statistics.**

| | Post-treatment $A\beta_{40}$ fibril | Pre-treatment $A\beta_{40}$ fibril |
|---|---|---|
| **Data collection** | | |
| Microscope | Titan Krios G2 | Titan Krios G2 |
| Voltage [keV] | 300 | 300 |
| Detector | K3 | K3 |
| Magnification | 81,000 | 81,000 |
| Pixel size [Å] | 1.05 | 1.05 |
| Defocus range [µm] | -0.7 to -2.4 | -0.7 to -2.4 |
| Exposure time [s/frame] | 3.0 | 2.95 |
| Number of frames | 40 | 40 |
| Total dose [$e^-$/Å$^2$] | ~40<br>(~1.0 $e^-$/Å$^2$/frame) | ~40<br>(~1.0 $e^-$/Å$^2$/frame) |
| **Reconstruction** | | |
| Micrographs | 7,311 | 21,576 |
| Box width [pixels] | 250 | 250 |
| Inter-box distance [pixels] | 13 | 13 |
| Picked segments (no.) | 3,193,361 | 9,147,664 |
| **Final map**[a] | | |
| Final segments [no.] | 326,836 | 888,252 |
| Final resolution [Å] (FSC=0.143) | 2.79 | 2.76 |
| Applied map sharpening B-factor [Å$^2$] | -114 | -121 |
| Symmetry imposed | C1 | C1 |
| Helical rise [Å] | 2.35 | 2.35 |
| Helical twist [°] | 179.65 | 179.65 |

[a] Sharpened map and refined atomic model are provided as SI files.

**Supplementary Table 4 | Model building statistics.**

| Lipid-induced PM | Post-treatment $A\beta_{40}$ fibril | Pre-treatment $A\beta_{40}$ fibril |
|---|---|---|
| **Initial model [PDB code]** | 8ovk | 8ovk |
| **Model composition**[a] | | |
| Chains | 10 | 10 |
| Non-hydrogen atoms | 3060 | 3060 |
| Protein residues | 400 | 400 |
| **RMS deviations** | | |
| Bond lengths [Å] | 0.02 | 0.01 |
| Bond angles [°] | 2.23 | 2.09 |
| **Validation** | | |
| MolProbity score | 1.68 | 1.70 |
| Clashscore | 5.86 | 6.19 |
| **Ramachandran plot** | | |
| Outliers [%] | 0 | 0 |
| Allowed [%] | 5.26 | 5.26 |
| Favored [%] | 94.74 | 94.74 |

[a] Sharpened map and refined atomic model l.
