# Supplementary material for: Anle138b binds predominantly to the central cavity in lipidic Aβ₄₀ fibrils and modulates fibril formation
Source: Nat Commun. 2025 Oct 3;16:8850. doi: 10.1038/s41467-025-64443-6 (PMC12494798; doi:10.1038/s41467-025-64443-6)
Supplement: Supplementary file 2 — Reporting Summary [file 41467_2025_64443_MOESM2_ESM.pdf]

nature portfolio

Corresponding author(s): C. Griesinger and L. B. Andreas

Last updated by author(s): Sep 4, 2025

Reporting Summary

Nature Portfolio wishes to improve the reproducibility of the work that we publish. This form provides structure for consistency and transparency in reporting. For further information on Nature Portfolio policies, see our Editorial Policies and the Editorial Policy Checklist.

Statistics

For all statistical analyses, confirm that the following items are present in the figure legend, table legend, main text, or Methods section.

n/a	Confirmed	
☐	☒	The exact sample size (*n*) for each experimental group/condition, given as a discrete number and unit of measurement
☐	☒	A statement on whether measurements were taken from distinct samples or whether the same sample was measured repeatedly
☒	☐	The statistical test(s) used AND whether they are one- or two-sided<br>*Only common tests should be described solely by name; describe more complex techniques in the Methods section.*
☒	☐	A description of all covariates tested
☒	☐	A description of any assumptions or corrections, such as tests of normality and adjustment for multiple comparisons
☐	☒	A full description of the statistical parameters including central tendency (e.g. means) or other basic estimates (e.g. regression coefficient) AND variation (e.g. standard deviation) or associated estimates of uncertainty (e.g. confidence intervals)
☒	☐	For null hypothesis testing, the test statistic (e.g. *F*, *t*, *r*) with confidence intervals, effect sizes, degrees of freedom and *P* value noted<br>*Give P values as exact values whenever suitable.*
☒	☐	For Bayesian analysis, information on the choice of priors and Markov chain Monte Carlo settings
☒	☐	For hierarchical and complex designs, identification of the appropriate level for tests and full reporting of outcomes
☒	☐	Estimates of effect sizes (e.g. Cohen's *d*, Pearson's *r*), indicating how they were calculated

Our web collection on statistics for biologists contains articles on many of the points above.

Software and code

Policy information about availability of computer code

Data collection	NMR: Bruker TopSpin(V3.6.5 and V4)<br>Cryo-EM :SerialEM(V4.0)<br>MD simulations: GROMACS 2023 (including implementations of P-LINCS, SETTLE, non-bonded Verlet scheme, PME, velocity-rescale Temperature coupling and Parrinello-Rahman barostat).
Data analysis	NMR data : CcpNMR(V 2.4.2), TopSpin(V 3.6.5).<br>ITC data : MicroCal control software.<br>Cryo-EM data : RELION(V3.1), CTFFIND(V4.1), COOT(V0.9), PHENIX(V1.19).<br>MD simulations: Awk and Bash scripts were used to postprocess output from GROMACS 2023 analysis tools (gmx mindist and gmx traj) and g_contacts (Blau et.al.) used to calculate interatomic distances.<br>Visualisation of fibril structure : Chimera(V1.8), ChimeraX(V1.9), Pymol

For manuscripts utilizing custom algorithms or software that are central to the research but not yet described in published literature, software must be made available to editors and reviewers. We strongly encourage code deposition in a community repository (e.g. GitHub). See the Nature Portfolio guidelines for submitting code & software for further information.

Data

Policy information about availability of data

All manuscripts must include a data availability statement. This statement should provide the following information, where applicable:

- Accession codes, unique identifiers, or web links for publicly available datasets
- A description of any restrictions on data availability
- For clinical datasets or third party data, please ensure that the statement adheres to our policy

NMR spectra raw data generated in this study of Amyloid beta (1-40) fibrils has been deposited in the BMRB under accession number 53129.
MD simulation input files, final coordinate files, and raw trajectory data generated in this study have been deposited in the Edmond data repository (https://doi.org/10.17617/3.NRYUVQ). Cryo-EM density maps and atomic models of L1 Aβ40 fibrils(both pre- and post-treatment fibril) have been deposited in the EMDB (EMD-53882 and EMD-53880) and PDB (9RAX and 9RAW).

Research involving human participants, their data, or biological material

Policy information about studies with human participants or human data. See also policy information about sex, gender (identity/presentation), and sexual orientation and race, ethnicity and racism.

Reporting on sex and gender	Not applicable
Reporting on race, ethnicity, or other socially relevant groupings	Not applicable
Population characteristics	Not applicable
Recruitment	Not applicable
Ethics oversight	Not applicable

Note that full information on the approval of the study protocol must also be provided in the manuscript.

Field-specific reporting

Please select the one below that is the best fit for your research. If you are not sure, read the appropriate sections before making your selection.

☒ Life sciences ☐ Behavioural & social sciences ☐ Ecological, evolutionary & environmental sciences

For a reference copy of the document with all sections, see nature.com/documents/nr-reporting-summary-flat.pdf

Life sciences study design

All studies must disclose on these points even when the disclosure is negative.

Sample size	Fibril preparation and characterization: Fibril samples were prepared and initially assessed by negative-stained electron microscopy (EM) to confirm fibril formation. Structural reproducibility was evaluated by solid-state NMR experiments (hCANH, hNCA, ^{13}C–^{13}C DARR, ^{13}C–^{13}C RFDR, and NHHC) using differently labeled fibrils: uniformly labeled with ^{13}C, uniformly labeled with ^{13}C and ^{15}N, perdeuterated (^{2}H, ^{13}C, ^{15}N), and selectively labeled with ^{13}C, ^{15}N at Lys and Ile residues.<br>NMR experiments: The number of scans was adjusted based on the signal-to-noise ratio (S/N). Data acquisition was continued until sufficient S/N was achieved.<br>Cryo-EM data acquisition: Cryo-EM data were collected for both pre-treatment and post-treatment conditions. The datasets comprised 21,576 micrographs for the pre-treatment fibrils and 7,311 micrographs for the post-treatment fibrils. Cryo-EM, NMR, and negative-stain EM measurements were all performed on the ^{1}H,^{13}C,^{15}N-labeled fibril sample.<br>MD simulations: a total of 20 MD simulations of anle138b binding to the fibril surface and the central fibril cavity were run for 250 to 1000 ns, respectively.
Data exclusions	No NMR data were excluded from the analysis.<br>Standard image classification procedures were employed to select particle images for high-resolution reconstructions, following established protocols (Scheres, J. Struct. Biol. 180, 519–530 (2021)). Details on the number of selected images are provided in Supplementary Table S3.
Replication	During fibril formation, samples were monitored using Thioflavin T (ThT) fluorescence, circular dichroism (CD) spectroscopy, and solid-state NMR (hCANH and hNH) experiments.<br>The protocol was repeated three times using differently labeled NMR samples ($^{1}H^{13}C^{15}N$, $^{2}H^{13}C^{15}N$, ^{13}C, and $^{13}C^{15}N$ Lys- or Ile-selectively labeled), consistently yielding identical spectra in both hCANH and hNH experiments.<br>Cryo-EM samples were validated using ThT fluorescence, CD spectroscopy, negative-stain EM, and NMR to confirm structural consistency with the fibril samples.<br>MD simulations: in all, for the different binding modes (fibril surface: with and without lipids; central cavity; protonated and unprotonated lysine 28), 40 simulations were run. Each condition was run between five to ten independent production simulations.

Randomization	Randomization was not performed for the both methods (ssNMR and Cryo-EM).
Blinding	Blinding was not applied for NMR experiments, as adjustment of experimental parameters requires prior knowledge of the isotope labeling scheme. Improper parameter settings could significantly increase measurement times, which range from several days to weeks.<br>Blinding was also not implemented for cryo-EM, as the risk of experimental bias was assessed to be minimal given the objective nature of image acquisition and particle selection procedures.

Reporting for specific materials, systems and methods

We require information from authors about some types of materials, experimental systems and methods used in many studies. Here, indicate whether each material, system or method listed is relevant to your study. If you are not sure if a list item applies to your research, read the appropriate section before selecting a response.

Materials & experimental systems

n/a	Involved in the study
☒	☐ Antibodies
☒	☐ Eukaryotic cell lines
☒	☐ Palaeontology and archaeology
☒	☐ Animals and other organisms
☒	☐ Clinical data
☒	☐ Dual use research of concern
☒	☐ Plants

Methods

n/a	Involved in the study
☒	☐ ChIP-seq
☒	☐ Flow cytometry
☒	☐ MRI-based neuroimaging

Plants

Seed stocks	Not applicable
Novel plant genotypes	Not applicable
Authentication	Not applicable
